# Impact of winemaking technologies on polyphenolic composition and wine microbiome

**DOI:** 10.3389/fmicb.2026.1846196

**Published:** 2026-05-08

**Authors:** Karolina Kostelnikova, Lucie Frejlichova, Milan Spetik, Jiri Sochor, Ales Eichmeier, Mojmir Baron

**Affiliations:** 1 Department of Viticulture and Oenology, Faculty of Horticulture, Mendel University in Brno, Lednice, Czechia; 2 Mendeleum–Department of Genetics, Faculty of Horticulture, Mendel University in Brno, Lednice, Czechia

**Keywords:** microbial succession, phenolic-microbial interaction, polyphenols, spontaneous/inoculated fermentation, wine microbiome

## Abstract

**Introduction:**

This study investigates how different oenological practices, including spontaneous and inoculated alcoholic fermentation (AF), variations in malolactic fermentation (MLF) and ageing, are associated with changes in microbial diversity and polyphenolic profile of Sauvignon blanc wines.

**Methods:**

Microbial composition was investigated through high-throughput DNA sequencing, while polyphenolic compounds were analysed using LC–MS together with total phenolic content through Folin–Ciocalteu assay and antiradical activity by DPPH assay.

**Results and discussion:**

AF was associated with a pronounced homogenization of the microbiota, particularly through the dominance of *Saccharomyces* and stable epiphytic bacteria. At later stages, microbial communities showed notable compositional divergence, with their development closely linked to technological interventions and the extent of phenolic extraction. The pomace-fermented treatment exhibited the highest polyphenol content along with the greatest compositional heterogeneity, whereas treatments with lower phenolic loads exhibited simpler microbial profiles and the stable dominance of *Leuconostoc* after malolactic fermentation. Polyphenols appear to act as modulatory factor in microbial succession, with extraction intensity showing a more distinct association with community shifts than the fermentation regime itself. Overall, the study highlights that technological practices affecting phenolic extraction appear to play a notable role in the observed microbial trends and the resulting wine characteristics.

## 1 Introduction

The microbiome of grapes, must, and wine is a complex community of yeasts and bacteria that play a fundamental role in shaping fermentation processes (Africa et al., 2025; Aires et al., 2025). Spontaneous fermentation, driven by the natural microbiota of the grapes and vineyard, potentially supplemented by microorganisms from the cellar environment, is characterized by high microbial diversity and a strong reflection of the terroir, whereas controlled fermentation using selected *Saccharomyces cerevisiae* strains proceeds in a more stable and predictable manner. In spontaneous fermentations, the early stages of alcoholic fermentation (AF) are typically dominated by non-*Saccharomyces* genera (e.g., *Hanseniaspora*, *Metschnikowia*, *Starmerella*, *Pichia*), while *Saccharomyces* predominates in the later stages as the principal producer of ethanol (Bekris et al., 2024; Fazio et al., 2025; González-Alonso et al., 2021; Granchi et al., 2019; Chen et al., 2022). An integral technological stage is malolactic fermentation (MLF), carried out by lactic acid bacteria (LAB), primarily of the genus *Oenococcus*, but also by certain species belonging to *Lactiplantibacillus* and *Levilactobacillus* (formerly grouped within *Lactobacillus*). Through the conversion of malic acid into lactic acid, these bacteria contribute to stabilization of the wine and significantly influence its sensory properties (Balmaseda et al., 2018).

Polyphenols are a broad group of secondary metabolites of grapevines that are essential in the development of wine color, sensory properties, antioxidant stability, and overall quality (Garrido and Borges, 2013; Gutiérrez-Escobar et al., 2021). They are transferred into the must and wine during grape processing, maceration, and fermentation, and their concentrations are influenced by the grape variety, terroir, degree of ripeness, and winemaking practices (Mitrović et al., 2024; Stoyanov et al., 2025). The polyphenolic profile of a wine is primarily composed of flavonoids, phenolic acids, and stilbenes (Garrido and Borges, 2013; Gutiérrez-Escobar et al., 2021).

There is a close bi-directional interaction between polyphenols and microorganisms (James et al., 2023; Piekarska-Radzik and Klewicka, 2021; Sabel et al., 2017; Tofalo et al., 2021). During fermentation, yeasts and bacteria modify phenolic compounds through enzymatic reactions that may result in hydrolysis, structural transformation, or alterations in antioxidant activity (James et al., 2023; Yang et al., 2023; Zhang et al., 2021; Zhao et al., 2025). Conversely, polyphenols influence microbial growth and metabolic activity and may selectively promote or inhibit specific microbial groups (Piekarska-Radzik and Klewicka, 2021; Sabel et al., 2017; Yang et al., 2023; Zhao et al., 2025). These interactions play a key role in shaping the aromatic profile, microbial stability, and biological value of wine (James et al., 2023; Minnaar et al., 2019; Tofalo et al., 2021). In addition, phenolic compounds can be adsorbed onto yeast cell walls or interact with mannoproteins released during yeast autolysis, affecting their concentration and reactivity in wine.

Winemaking technology has a profound influence on both microbial communities and the polyphenolic profile of wine (Branco et al., 2025; Englezos et al., 2022). Spontaneous and controlled AF differ in their microbial dynamics and fermentation progression (Bagheri et al., 2020; Živković et al., 2024) while different MLF regimes modify the acid profile, wine stability, and formation of secondary metabolites (Ghanem et al., 2014; Minnaar et al., 2019). Filtration can reduce both microorganisms and their metabolites, thereby affecting the phenolic composition of filtered and unfiltered wines (Ghanem et al., 2014; Mazauric and Salmon, 2005). In addition, ageing conditions, lees contact, and vessel type further influence microbial activity as well as the extraction and transformation of polyphenols, ultimately shaping the overall character of the wine (Branco et al., 2025; Mazauric and Salmon, 2005). However, existing literature indicates that microbial succession is a multifactorial process influenced by multiple interacting factors, including ethanol, pH, temperature, and SO₂ levels, which may modulate or outweigh individual technological effects (James et al., 2023; Windholtz et al., 2025; Wu et al., 2026).

While individual oenological practices are well-documented, integrated research on how diverse vinification trajectories simultaneously shape the wine microbiome and phenolic profile remains limited. We hypothesize that prolonged skin-contact and specific MLF regimes play a major role in shaping these changes, potentially exerting a stronger influence on the final wine profile than the initial fermentation type (spontaneous vs. inoculated). To test this hypothesis, we investigated how different vinification practices (including spontaneous and controlled AF, contrasting MLF regimes, and variations in grape processing) and the subsequent evolution during storage period are associated with changes in the microbial communities and polyphenolic profile of Sauvignon blanc wines. This variety was selected due to its global economic significance and its high sensitivity to phenolic extraction and oxygen management, making it an ideal model for evaluating the impact of different vinification strategies on wine stability. By focusing on the relationship between microbial succession and changes in phenolic composition, this study utilizes high-throughput DNA sequencing alongside LC–MS analysis, total phenolic content determination, and antioxidant activity assays. This integrative approach enabled a direct comparison of the individual technological treatments and provided a deeper insight into how fermentation-related processes influence the dynamics of microorganisms and phenolic compounds during winemaking.

## 2 Materials and methods

### 2.1 Material

In 2024 grapes of the Sauvignon blanc cultivar were harvested by hand from the Na Valtické vineyard in the wine-growing village of Lednice (Mikulov sub-region, Moravia wine region, Czech Republic) for use in the experiment. The must had the following basic parameters: sugar content 22.7 °Bx, total acidity 6.42 g/L, pH 3.45, and yeast assimilable nitrogen content 189 mg/L.

### 2.2 Experimental design and vinification

Grapes of the Sauvignon cultivar were harvested by hand and processed immediately after harvest. Following destemming and crushing, a portion of the pomace was separated prior to pressing and used for treatment V7 (M-S/S), which underwent spontaneous AF and spontaneous MLF directly on the pomace (skins and seeds). The remaining pomace was subsequently pressed using a pneumatic press.

Immediately after pressing, a portion of the fresh, non-settled must was divided into three batches corresponding to treatments V1–V3:

- V1 – NS-S/S: spontaneous AF + spontaneous MLF
- V2 – NS-S/C: spontaneous AF + controlled MLF
- V3 – NS-S/N: spontaneous AF + no MLF

These treatments were prepared from non-settled must and all underwent spontaneous AF. Treatment V1 was subjected to spontaneous MLF, Treatment V2 to controlled MLF, while MLF was suppressed in Treatment V3.

The remaining portion of the must was allowed to settle through static sedimentation for 24 h at 6–8 °C. On the following day, the clarified must was racked off the sediment and divided into treatments V4–V6:

- V4 – S-C/S: controlled AF + spontaneous MLF
- V5 – S-C/C: controlled AF + controlled MLF
- V6 – S-C/N: controlled AF + no MLF

**Figure 1:**
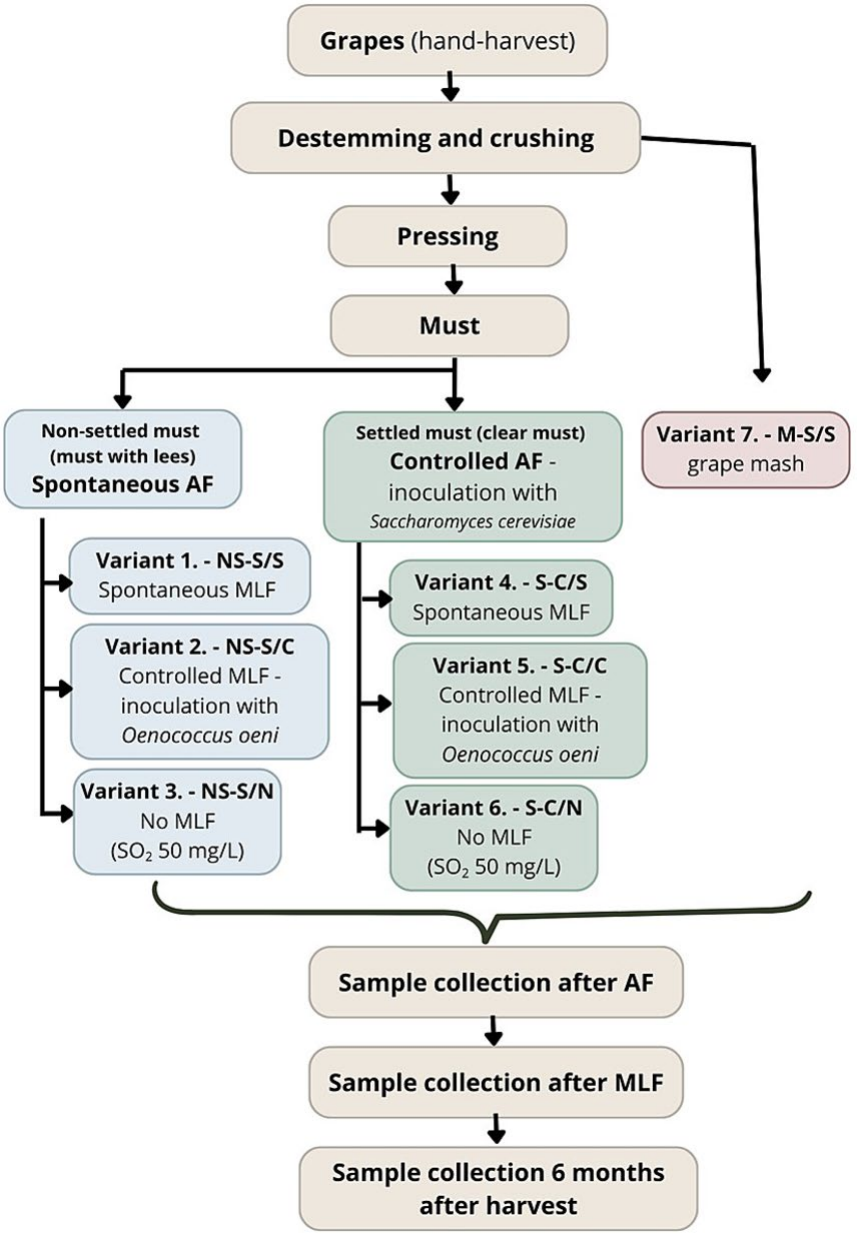
Experimental process. NS, Non-settled must; S, Settled must; M, Pomace fermentation; S, Spontaneous fermentation (AF or MLF); C, Controlled fermentation (AF or MLF); N, no MLF (SO_2_ 50 mg/L).

These treatments underwent controlled AF following inoculation with *Saccharomyces cerevisiae* (IOC Révélation Terroir, Institut Œnologique de Champagne, France). The course of MLF followed the experimental design: V4 underwent spontaneous MLF, V5 controlled MLF, and V6 no MLF. Treatment V7 (M-S/S) was produced separately, with both spontaneous AF and spontaneous MLF taking place directly on the pomace (skins and seeds). The use of non-settled must for spontaneous treatments (V1–V3) and clarified must for inoculated treatments (V4–V6) was selected to reflect authentic winemaking practices. This design preserves indigenous microbiota in spontaneous fermentations while following standard industrial protocols for inoculated batches. All seven experimental treatments (V1–V7) were prepared in triplicate, with each replicate fermented in a 30-L glass fermentation vessel (see Figure 1).

After completion of AF, samples were collected from all treatments. Treatments designated for controlled MLF (V2 and V5) were inoculated with a commercial *Oenococcus oeni* strain (Viniflora Oenos 2.0, Chr. Hansen A/S, Denmark). In treatments without MLF (V3 and V6), sulfur dioxide was added at a dose rate of 50 mg/L. Following fermentation, the wines were racked off the gross lees and returned to the vessels. To ensure they were filled to capacity (minimal headspace), the vessels were topped up with reserve wine from the same treatment batch, produced in parallel under identical conditions. The wines were then stored until the final sampling 6 months after harvest. The progression of AF and MLF was monitored via internal quality control using FTIR spectroscopy for residual sugar and HPLC for organic acid analysis. All fermentations proceeded successfully, with all treatments reaching dryness by the end of the six-month maturation period. Residual sugar concentrations at the final stage were below 0.5 g/L. Sampling stages included: must prior to fermentation (non-settled and settled), after AF, after MLF, and 6 months after harvest.

### 2.3 Microbiome analysis

The microbial composition of the samples was analyzed using massively parallel sequencing (next-generation sequencing, NGS). Prior to DNA extraction, samples from the three technological replicates of each treatment and sampling point were pooled in equal proportions to obtain a single composite sample. The samples (50 mL) were then centrifuged at 16,000 x *g* for 15 min and the resulting pellet was used in the subsequent processing. Genomic DNA was isolated using the NucleoSpin® Tissue kit (Macherey-Nagel, Düren, Germany) according to the manufacturer’s instructions. Following extraction, DNA concentration and quality underwent spectrophotometric analysis. Following quantification, the DNA was normalized to a final concentration of 10 ng/μL.

#### 2.3.1 PCR amplification of target regions

PCR amplicons were generated to target the following genomic regions: the ITS (Internal Transcribed Spacer) region for fungi, including yeasts and filamentous fungi, and the V4 region of the bacterial 16S rRNA gene (approximately 300 bp).

PCR reactions were prepared as a master mix according to the manufacturer’s protocol (Promega, Madison, WI, United States). The reaction mixture contained HPLC-grade water, GoTaq® Reaction Buffer, MgCl₂, dNTPs (Thermo Fisher Scientific, Waltham, MA, United States), GoTaq® Green dye, GoTaq® DNA Polymerase (Promega, Madison, WI, United States), and target-specific primers.

The following primer pairs were used:

- Fungi (ITS) (Bokulich and Mills, 2013):
  - Forward: BITS (ACCTGCGGARGGATCA)
  - Reverse: B58S3 (GAGATCCRTTGYTRAAAGTT)
- Bacteria (16S rRNA) (Klindworth et al., 2013):
  - Forward: S-D-Bact-0008-a-S-16 (GACTACHVGGGTATCTAATCC)
  - Reverse: S-D-Bact-0785-a-A-21 (AGAGTTTGATCMTGGCTCAG)

Following amplification, PCR products were purified using the NucleoSpin® Gel and PCR Clean-up kit (Macherey-Nagel, Düren, Germany) to remove residual reagents. Purified DNA was quantified fluorometrically and diluted to a final concentration of 10 ng/mL for sequencing library preparation.

#### 2.3.2 Preparation of sequencing libraries and sequencing

Sequencing libraries were prepared using the Nextera XT DNA Library Prep Kit (Illumina, San Diego, CA, USA) according to the standard Illumina protocol.

Sequencing was performed using Illumina Sequencing by Synthesis (SBS) technology on a MiniSeq platform (Illumina, San Diego, CA, United States). Following library loading, cluster generation was carried out on the flow cell surface, after which sequencing-by-synthesis cycles were executed according to the manufacturer’s specifications.

#### 2.3.3 Bioinformatic data processing

The sequencing data were processed using the SEED v2.1.2 software environment. Initial quality control was performed using FastQC v0.10.1 (Andrews, 2010), followed by adapter removal and quality filtering. Paired-end reads were merged using fastq-join (Aronesty, 2013), and sequences shorter than 70 bp or containing ambiguous bases were discarded. Only reads with an average Phred quality score ≥ Q30 were retained for downstream analysis. For fungal datasets, the ITS2 region was extracted using ITSx v1.1.2 (Bengtsson-Palme et al., 2013).

Operational Taxonomic Units (OTUs) were clustered at a 97% similarity threshold using USEARCH v8.1.1861 (Blaxter et al., 2005). Singleton reads and chimeric sequences were identified and removed using the UPARSE algorithm within USEARCH (Edgar, 2013). Taxonomic assignment was carried out using BLASTn (minimum identity 98%, maximum e-value 1e−50) against the UNITE fungal database (v8.2) and the SILVA bacterial database (v138) (Nilsson et al., 2019; Quast et al., 2012). Sequences failing to meet these taxonomic criteria or matching non-target organisms were excluded from the final datasets (Baldrian et al., 2022; Nilsson et al., 2019).

### 2.4 Determination of individual polyphenolic components

A total of 31 polyphenolic compounds belonging to the main groups of flavonols, hydroxybenzoic acids, hydroxycinnamic acids, stilbenes, and flavan-3-ols were analyzed in the samples. These compounds were selected based on a targeted approach, utilizing a validated in-house LC–MS method optimized for these specific analytes, which serve as representative markers for monitoring the phenolic evolution in white wines. Detailed information on the detected compounds and their classification is provided in Section 3.3 (Table 1).

**Table 1:** Individual polyphenolic components.

Polyphenol group	Individual compounds
Flavonols	Myricetin*, quercetin, quercetin-3-glukosides, rutin*, kaempferol*, kaempferol-3-D-glukoside*, isorhamnetin*
Hydroxybenzoic acids	Gallic acid, protocatechuic acid, 4-hydroxybenzoic acid
Hydroxycinnamic acids	Caftaric acid, GRP (grape reaction product or 2-S-glutathionyl caftaric acid), caffeic acid, ethylcaffeate, coutaric acid, *p*-coumaric acid, ethylcoumarate, fertaric acid, ferulic acid
Stilbenes	*Trans*-resveratrol, *cis*-resveratrol, *trans*-piceid, *cis*-piceid, piceatannol, astringin
Flavan-3-ols and procyanidins	Catechin, epicatechin, epicatechin-3-gal*, procyanidin B1, procyanidin B2, procyanidin C*

*Compounds remained below the detection limit across all treatments.

The concentrations of individual phenolic compounds were analyzed using an LC–MS method with direct sample injection. Prior to analysis, the wine samples were centrifuged at 3000 g for 6 min. Must and wine samples were prepared by mixing 500 μL of sample, 20 μL of internal standard solution, and 480 μL of 10% formic acid. After homogenization, the samples were centrifuged again (3,000 g for 6 min), and 750 μL of the supernatant was transferred to autosampler vials for subsequent analysis.

The internal standard consisted of a mixture of 2 mM HCCA (*α*-cyano-4-hydroxycinnamic acid) and 2 mM trolox ((±)-6-hydroxy-2,5,7,8-tetramethylchroman-2-carboxylic acid). LC–MS analyses were performed using a binary high-pressure ExionLC™ AC system (online degasser, dual pumps, autosampler, column oven, and control unit) coupled to a Sciex QTrap 3,200 mass spectrometer.

Chromatographic separation was performed on an Arion Polar C18 column (2.2 μm; 2.1 × 100 mm) maintained at 60 °C. The injection volume was 5 μL and the mobile phase flow rate was set to 0.3 mL/min. Mobile phase A consisted of water containing 1% formic acid (HCOOH), while mobile phase B was acetonitrile containing 1% HCOOH. Gradient elution was applied as follows: 0.00 min, 6% B; 3.00 min, 9% B; 6.00 min, 12% B; 9.00 min, 18% B; 12.00 min, 30% B; 15.00 min, 60% B; 16.00 min, 90% B; 16.01–16.99 min, 0% B; and 17.00 min, return to 6% B. The total run time, including re-equilibration, was 20 min. The mass spectrometer recorded chromatographic data within the retention time window of 0.9–16 min. Quantification of the individual phenolic compounds was carried out using external calibration curves constructed from the corresponding analytical standards (Sigma-Aldrich, St. Louis, MO, United States).

The QTrap 3,200 mass spectrometer was operated in multiple reaction monitoring (MRM) mode. Electrospray ionization (ESI) was performed in negative ion mode with an ion spray voltage of −4,200 V. The gas parameters were set as follows: curtain gas (CUR) 45 psig, collision gas (CAD) at medium intensity, nebuliser gas (GS1) 60 psig, and turbo gas (GS2) 45 psig. The desolvation temperature was maintained at 600 °C. MS/MS transitions and compound-specific parameters for individual analytes are listed in Supplementary Table S1.

### 2.5 Determination of total phenolic content

Total phenolic content (TPC) was determined using a modified Folin–Ciocalteu method (Waterman and Mole, 1994). Prior to analysis, the must and wine samples were centrifuged at 3000 *g* for 6 min. An aliquot of 980 μL of a 3% sodium carbonate decahydrate solution was transferred into a 1.5 mL Eppendorf tube, followed by the addition of 20 μL of sample and 50 μL of Folin–Ciocalteu reagent. The mixture was homogenized and incubated for 120 min in the dark at room temperature. Absorbance was measured at 750 nm against a reagent blank. Total phenolic concentration was calculated using a gallic acid calibration curve (25–1,000 mg/L) and expressed as mg/L gallic acid equivalents (GAE).

### 2.6 Determination of antiradical activity–DPPH assay

Prior to analysis, all wine samples were centrifuged at 3000 g for 6 min. An aliquot of 980 μL of a 200 μM solution of DPPH (2,2-diphenyl-*β*-picrylhydrazyl) in methanol was transferred into a 1.5 mL tube, followed by the addition of 20 μL of sample. The mixture was homogenized and incubated for 30 min at room temperature in the dark. Absorbance was measured at 515 nm against a reagent blank. Antiradical activity was calculated from the difference between the absorbance of the blank and the sample and quantified using a calibration curve prepared with gallic acid (10–200 mg/L). The results are expressed as mg/L gallic acid equivalents (GAE).

### 2.7 Statistical analysis

Statistical analyses were performed using Statistica 14 software (TIBCO Software Inc.) and the R environment (version 4.3). Each technological treatment was produced in three independent replicates. For polyphenolic analyses, each replicate was analyzed in triplicate; reported values therefore represent mean values calculated from nine measurements per treatment and sampling point. For must samples, mean values were calculated from three analytical measurements. The results are shown in the corresponding figures. For TPC, DPPH, and the individual polyphenolic compounds, the differences between the technological treatments at the final sampling point (6 months) were evaluated using Tukey’s HSD test (*α* = 0.05). Mean values were subsequently assigned to homogeneous groups denoted by letters (a, b, c, …), which are displayed directly in the figures. For TPC and DPPH, a two-way (factorial) analysis of variance (ANOVA) was additionally applied with technological treatment and fermentation stage as fixed factors including their interaction. The relationship between TPC and AA was assessed using Spearman’s rank correlation.

For microbial community analyses, biological replicates were pooled into a single composite sample per treatment and sampling point to obtain a representative overview of the microbial community. This pooling procedure precludes formal statistical comparisons between treatments at the microbiome level. Consequently, microbial diversity (Shannon index) and composition (PCoA, Heatmaps) were analyzed descriptively to identify major trends and shifts between treatments. Beta diversity was visualized through principal coordinates analysis (PCoA) based on Bray–Curtis dissimilarity. Prior to ordination, abundance data were transformed using centred log-ratio (CLR) transformation. Heatmaps were generated using hierarchical clustering (Ward’s method, Bray–Curtis distance). The core microbiome was defined as taxa present in ≥ 50% of samples with a minimum relative abundance of 0.01%. This threshold was used to identify stable community members and filter out transient or rare taxa.

## 3 Results and discussions

In the following sections, it should be noted that treatment V7 (pomace-fermented Sauvignon blanc) represents a fundamentally distinct vinification system compared to the other variants. Given its intensive skin contact, V7 serves primarily as a high-extraction reference point to evaluate microbial responses under conditions of elevated phenolic load, rather than a treatment intended for direct statistical comparison with the standard must-based vinifications (V1–V6).

### 3.1 Microbial communities during vinification

#### 3.1.1 Fungal communities (yeast and filamentous fungi)

**Figure 2:**
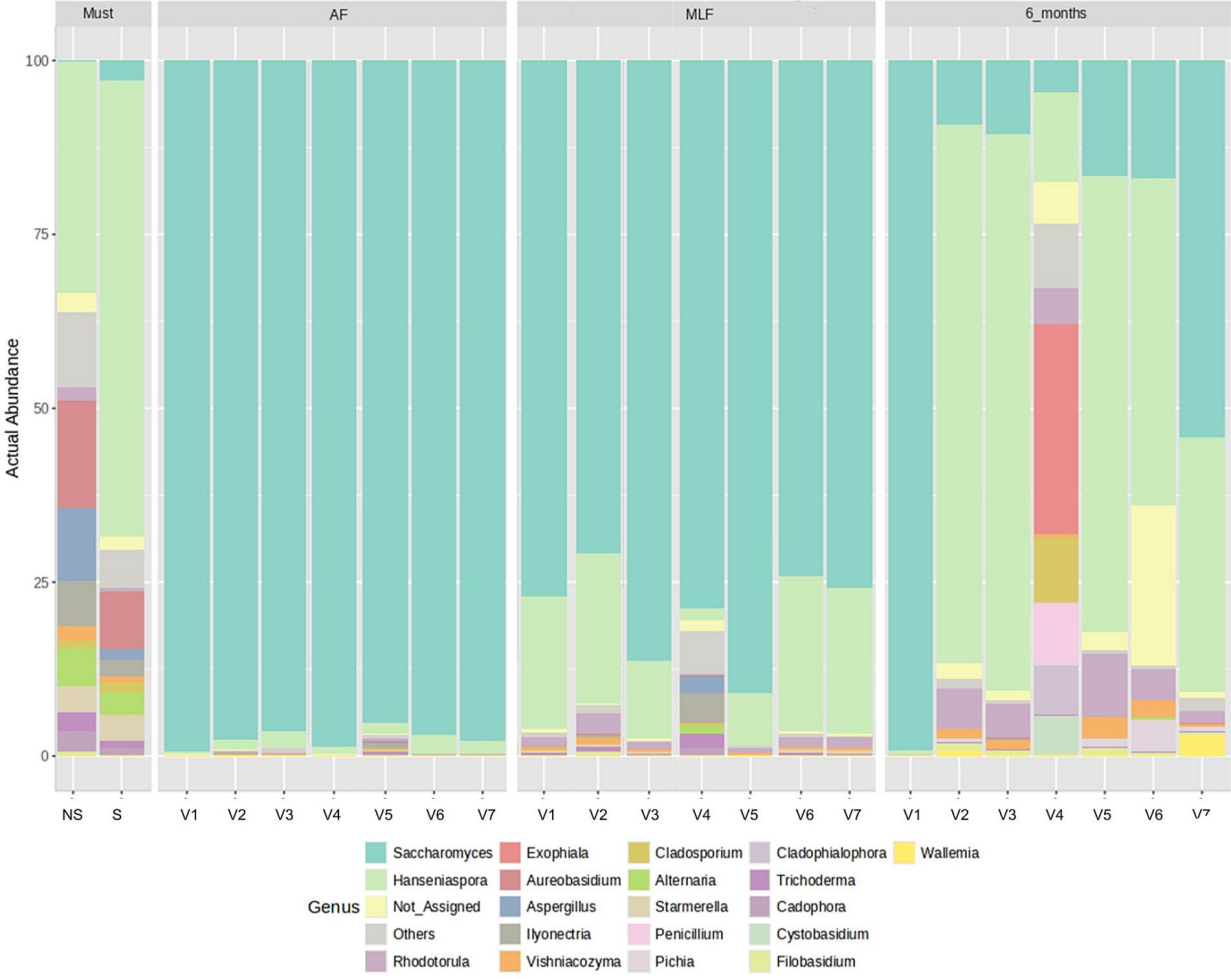
Relative abundance profiles of fungal genera across technological treatments and fermentation stages. V1 (NS-S/S), V2 (NS-S/C), V3 (NS-S/N), V4 (S-C/S), V5 (S-C/C), V6 (S-C/N), and V7 (M-S/S). NS, Non-settled must; S, Settled must; M, Pomace fermentation; S, Spontaneous fermentation (AF or MLF); C, Controlled fermentation (AF or MLF); N, no MLF (SO₂ 50 mg/L).

Reflecting real-world technological scenarios, the initial mycobiome appeared to be associated with the specific must preparation (settled vs. non-settled) used for the respective fermentation strategies. As shown in Figure 2, the composition of the fungal microbiome across these samples followed the successional patterns described in recent literature. In must, the microbial diversity was high and was made up of a broad spectrum of epiphytic species, dominated by the genera *Hanseniaspora*, *Rhodotorula*, *Aureobasidium*, *Cladosporium*, *Alternaria*, *Aspergillus*, *Penicillium*, and *Trichoderma*. This type of community structure is characteristic of the early stages of vinification and has been consistently reported by Shimizu et al. (2023), Garau et al. (2025), Wei et al. (2018), and De Celis et al. (2022). Clarification of the must by sedimentation was associated with a notable reduction in microbial diversity, in line with well-established observations that clarification efficiently removes a substantial proportion of grape- and cellar-associated microflora.

Following AF, all treatments exhibited an almost identical microbial profile dominated by the genus *Saccharomyces*, whereas other yeasts and filamentous fungi were detected at trace levels. This compositional shift is a typical feature of AF, during which *Saccharomyces* rapidly outcompetes non-*Saccharomyces* yeasts owing to its high ethanol tolerance and strong competitive capacity. Similar dominance patterns during AF have been widely documented by Shimizu et al. (2023), Bagheri et al. (2020), Zott et al. (2010), and Martins et al. (2024).

After MLF, *Saccharomyces* remained the dominant genus in all treatments. However, the overall structure of the mycobiome began to diverge compositionally among the treatments. Treatments V3 and V5 exhibited the highest relative abundance of *Saccharomyces* and a profile approaching monodominance, whereas V1, V2, V6, and V7 displayed very similar community compositions characterized by a dominant *Saccharomyces* population accompanied by a minor contribution of other taxa, particularly *Hanseniaspora*. A notable divergence was observed in V4, which showed increased mycobiome diversity with a more substantial representation both yeasts, (e.g., *Hanseniaspora and Starmerella*) and filamentous fungi or yeast-like genera *Aureobasidium*, *Cladosporium*, *Alternaria*, *Trichoderma*, *Cadophora, and Ilyonectria*. These observations align with reports that indicate individual non-*Saccharomyces* fungi differ in their ecological tolerance and may persist or re-emerge depending on the technology used and the course of fermentation (De Celis et al., 2022; Garau et al., 2025; Prathiviraj et al., 2022).

After 6 months, mycobiome profiles showed further descriptive divergence among the treatments. V1 remained largely monocultural, with a continued dominance of *Saccharomyces*. V2, V3, V5, and V6 showed highly similar compositions characterized by the predominance of *Hanseniaspora* alongside *Saccharomyces*. V7 maintained a relatively higher abundance of *Saccharomyces*, accompanied by *Hanseniaspora* and a more pronounced occurrence of *Wallemia*. Again, a distinct profile was observed in V4, in which the relative abundance of *Saccharomyces* and *Hanseniaspora* declined markedly, accompanied by an increase in other fungal taxa, particularly *Exophiala*, with the presence of *Rhodotorula*, *Cladosporium*, *Penicillium*, *Cladophialophora*, and *Cystobasidium*. This pattern corresponds to the highest compositional heterogeneity observed after ageing. Similar recolonization processes during the ageing of wine have been reported by De Celis et al. (2022) and Martins et al. (2024), who attributed these shifts to the late stages of vinification which are no longer ecologically dominated by the fermentative activity of *Saccharomyces*, thereby allowing less competitive or environment-adapted species to become re-established.

Overall, the results suggest that the dynamics of the yeast microbiome in the analyzed wines follow the general ecological patterns of fermentation, characterized by high initial diversity, rapid selection in favor of *Saccharomyces* during AF, technology-related differences in minor taxa during MLF, and a gradual divergence of fungal communities during ageing.

##### 3.1.1.1 Alpha and beta diversity of fungal communities

**Figure 3:**
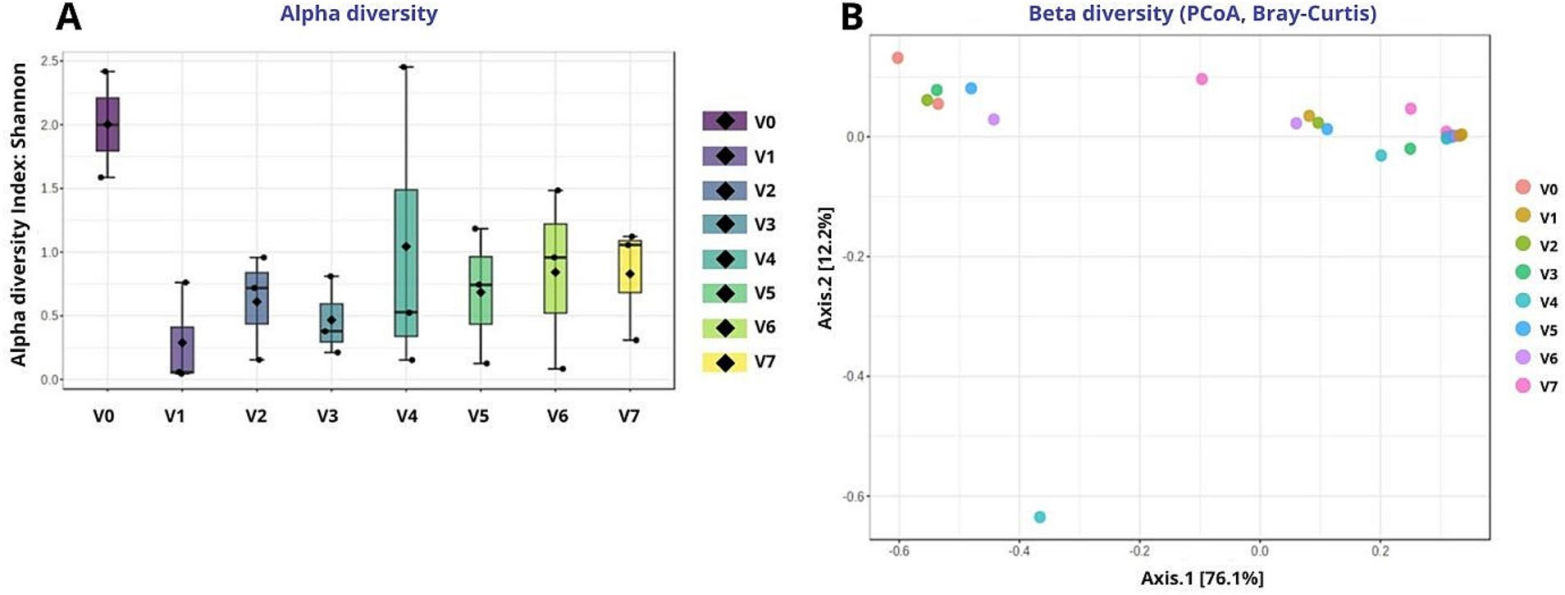
Diversity pattern of fungal communities across different technological treatments. **(A)** Alpha diversity (Shannon index); **(B)** beta diversity visualized by PCoA on Bray–Curtis dissimilarity. Treatments codes: V0 (Must), V1 (NS-S/S), V2 (NS-S/C), V3 (NS-S/N), V4 (S-C/S), V5 (S-C/C), V6 (S-C/N), and V7 (M-S/S). NS, Non-settled must; S, Settled must; M, Pomace fermentation; S, Spontaneous fermentation (AF or MLF); C, Controlled fermentation (AF or MLF); N, No MLF (SO₂ 50 mg/L).

Alpha diversity analysis (Shannon index; Figure 3A) suggested a sharp decrease in fungal diversity after the onset of AF across all treatments. The highest diversity was observed in must, reflecting the naturally rich assemblage of grape-associated and environmental fungi. Upon initiation of AF, diversity decreased sharply in all treatments due to the dominance of *Saccharomyces*, with a comparable decline regardless of fermentation strategy, reflecting strong selective pressure associated with ethanol accumulation, low pH, and anaerobic conditions. During MLF and subsequent ageing, only minor differences in diversity were observed among treatments. Slightly higher contributions of minor taxa were detected in some treatments (e.g., V4 and V6), suggesting possible limited recolonization or persistence of environmental fungi, while V7 exhibited intermediate diversity levels. Overall, these descriptive trends suggest that fungal alpha diversity was primarily linked to the fermentation stage rather than by the specific fermentation or MLF regime.

Beta diversity analysis visualized through PCoA based on Bray–Curtis dissimilarity (see Figure 3B) indicated that fungal community composition was primarily structured by fermentation stage. Must samples formed a distinct cluster reflecting their high complexity, whereas all samples collected after the onset of AF clustered tightly due to the near-complete dominance of *Saccharomyces*. Samples obtained after MLF largely overlapped, suggesting that the MLF regime had only a minor notable effect on overall mycobiome structure. A slight separation of the pomace-fermented treatment V7 was observed, which appears to be associated with a higher contribution of minor taxa. In summary, fungal beta diversity trends were mainly related to fermentation progression, with technological differences playing a secondary role.

##### 3.1.1.2 Core fungal microbiome

Analysis of the core microbiome (see Supplementary Figure S1) suggested that *Saccharomyces* was the only genus consistently present across all samples, maintaining a high prevalence even under more stringent abundance thresholds. *Hanseniaspora* was consistently detected at low abundance thresholds, and its occurrence was largely confined to the early fermentation stages, consistent with its role as an early but transient community member. All other genera exhibited low stability as thresholds increased, suggesting that they do not constitute a stable component of the core microbiota. This analysis complements the abundance-based results by focusing on the persistence of taxa across samples rather than their relative abundance. Consequently, the core mycobiome of the analyzed wines appears to be very simple and was likely shaped by the strong selective pressure exerted by *Saccharomyces* during fermentation.

##### 3.1.1.3 Abundance patterns and hierarchical clustering (heatmap analysis)

**Figure 4:**
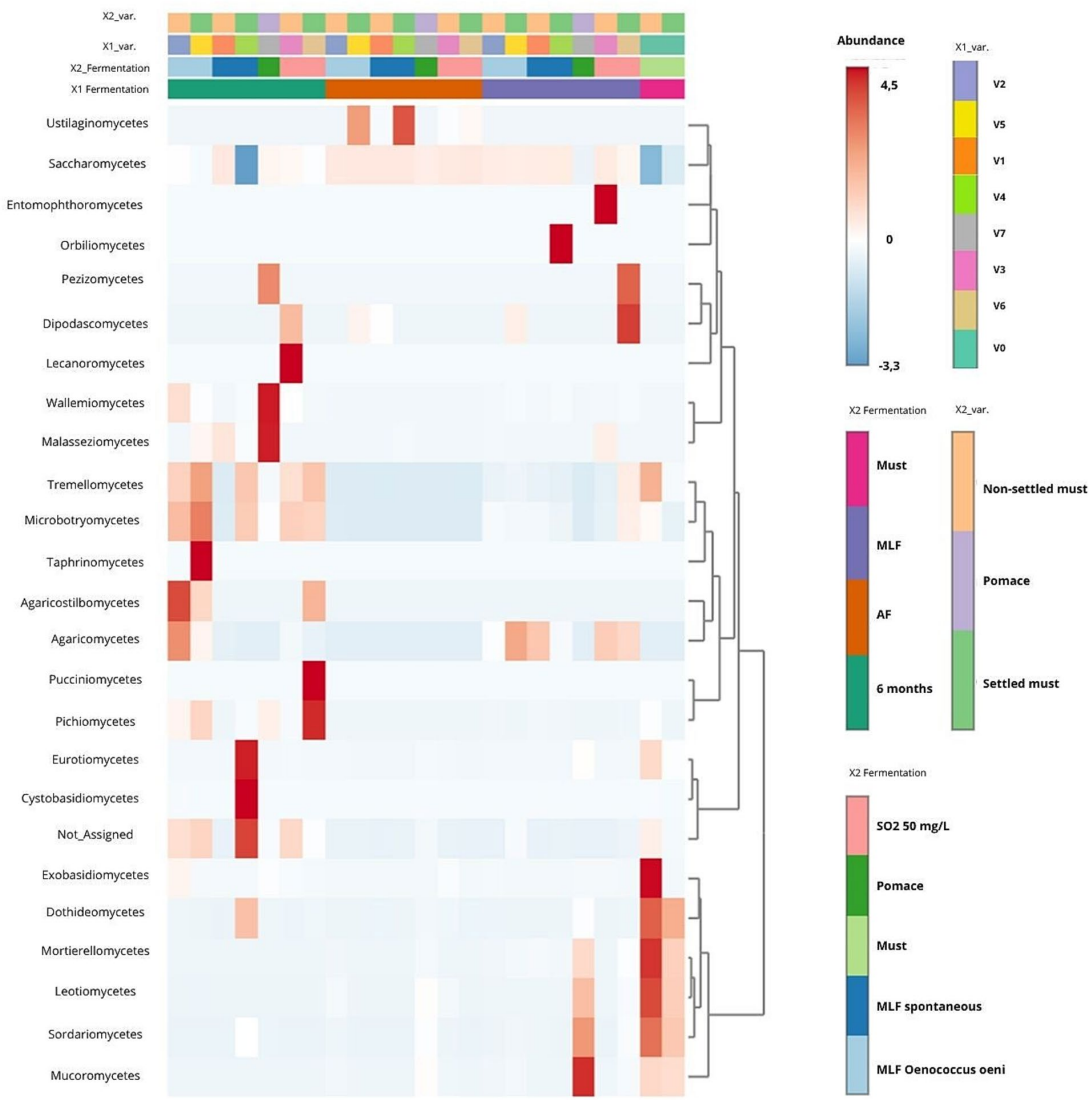
Taxonomic composition and hierarchical clustering of fungal communities across different technological treatments. Treatment codes: V0 (Must), V1 (NS-S/S), V2 (NS-S/C), V3 (NS-S/N), V4 (S-C/S), V5 (S-C/C), V6 (S-C/N), and V7 (M-S/S). NS, Non-settled must; S, Settled must; M, Pomace fermentation; S, Spontaneous fermentation (AF or MLF); C, Controlled fermentation (AF or MLF); N, No MLF (SO_2_ 50 mg/L).

Heatmap analysis at the class level (see Figure 4) suggested a strong selective shaping of the mycobiome during vinification, providing a macro-taxonomic overview that complements the genus-level succession shown in Figure 2. Must samples showed high taxonomic diversity, whereas alcoholic fermentation was associated with the dominance of *Saccharomycetes*, which persisted through malolactic fermentation and ageing. Technological interventions, including sulphur dioxide addition and *Oenococcus oeni* inoculation, appeared to further reduce compositional heterogeneity and a higher contribution of environmental taxa.

Hierarchical clustering indicated that the primary separation occurred between must and fermented samples, with compositional variations among technological treatments being of secondary importance. Overall, the heatmap highlights progressive simplification of the mycobiome during vinification, alongside increased taxonomic variation associated with extractive practices, particularly in the skin-contact treatment V7. This high-level clustering is consistent with the observation that the fermentation stage is the primary factor structuring the fungal community structure, while specific oenological practices further refine the presence of minor taxa.

#### 3.1.2 Bacterial communities

**Figure 5:**
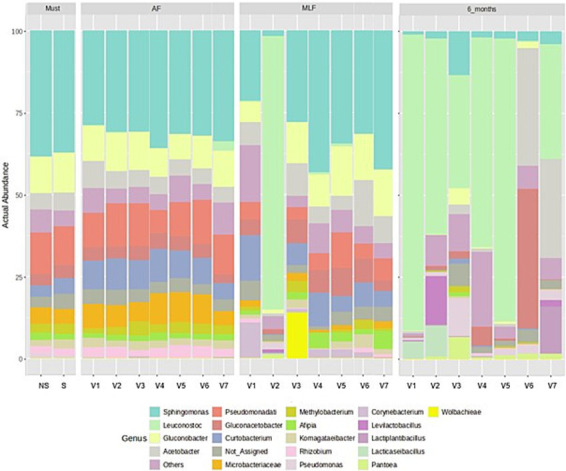
Relative abundance profiles of bacterial genera across technological treatments and fermentation stages. V1 (NS-S/S), V2 (NS-S/C), V3 (NS-S/N), V4 (S-C/S), V5 (S-C/C), V6 (S-C/N), and V7 (M-S/S). NS, Non-settled must; S, Settled must; M, Pomace fermentation; S, spontaneous fermentation (AF or MLF); C, Controlled fermentation (AF or MLF); N, No MLF (SO₂ 50 mg/L).

The bacterial profile of the must reflected the typical epiphytic microbiome of grapes (see Figure 5), which is commonly composed of genera such as *Sphingomonas*, *Gluconobacter*, *Acetobacter*, *Pseudomonas*, and members of the family *Microbacteriaceae*, while LAB are rarely detected at this stage. This pattern is well documented in the literature, which consistently reports that the primary bacterial community associated with grape surfaces is dominated by aerobic and soil-epiphytic bacteria rather than LAB (Aires et al., 2025; Bouki et al., 2025; García-Izquierdo et al., 2024; Hamaoka et al., 2022; Kamilari et al., 2021). Must clarification is generally reported to reduce yeast diversity more strongly than it does bacterial diversity. The descriptive trends observed in the present study appear to be consistent with these reports (Aires et al., 2025).

Following AF, bacterial community structure showed a trend toward homogenization across all treatments. This shift aligns with common outcome of AF, which generates an environment characterized by elevated ethanol concentrations, low pH, and limited oxygen availability, thereby selecting for acetic acid bacteria and a small number of epiphytic taxa while suppressing most other bacterial groups. After AF the dominant taxa included *Sphingomonas*, *Pseudomonadati*, *Gluconobacter*, *Curtobacterium*, and other aerobic or facultatively anaerobic bacteria originating from grape surfaces and the winery environment. Similar compositional patterns following AF have been reported in studies investigating both spontaneous and inoculated fermentation (García-Izquierdo et al., 2024; Liang et al., 2023; Mesnage et al., 2025). A notable observation was made in V7, in which the genus *Leuconostoc* was detected after AF, unlike the other treatments. This observation is consistent with previous findings indicating that maceration-based and pomace fermentation technologies may promote increased bacterial abundance due to higher nutrient release from grape solids (Bouki et al., 2025; Li et al., 2021).

After MLF, bacterial community profiles showed notable compositional divergence, likely reflecting both the MLF regime and the winemaking technology. The inoculated V2 exhibited the expected presence of *Leuconostoc*, sometimes accompanied by *Levilactobacillus*, a pattern characteristic of controlled MLF (Filimon et al., 2022; Paramithiotis et al., 2022). In contrast, spontaneous MLF (V1 and V4) was characterized by more variable successional trajectories. In V1, an increase in *Corynebacterium* was observed, while V4 remained compositionally similar to the post-AF state. These patterns align with the high variability reported for spontaneous MLF, which is strongly shaped by the initial microbial composition of the wine (Filimon et al., 2022; Manera et al., 2023; Rivas et al., 2022). Distinct ecological trajectories were observed in the treatments in which MLF was suppressed. Treatment V3 was characterized by the dominance of members of *Wolbachieae*, whereas V6 retained a community structure similar to that observed after AF, with a moderate decline in *Microbacteriaceae*. These profiles are consistent with the behavior of wines lacking biological stabilization (Aires et al., 2025; James et al., 2023). V7 exhibited an increase in *Sphingomonas* without detectable LAB, suggesting an alternative successional pathway associated with fermentation on grape skins.

After 6 months, the bacterial community profiles showed futher descriptive divergence, with *Leuconostoc* dominating in most samples. This genus is known for its ability to persist after MLF, even at low sulphur dioxide concentrations (Rivas et al., 2022). The dominance of *Leuconostoc* was most pronounced in V1 and V5. In contrast, treatment V2 exhibited a broader LAB assemblage, consistent with the higher LAB diversity commonly observed following inoculated MLF (Paramithiotis et al., 2022). In treatments without MLF, V3 showed increase in *Pantoea* and *Pseudomonas*, while V6 was dominated by acetic acid bacteria, particularly *Acetobacter*, which are capable of long-term persistence under conditions unfavorable for LAB growth (Sun et al., 2022). V7 displayed increased proportions of *Leuconostoc*, *Acetobacter*, and *Lactiplantibacillus*, suggesting enhanced bacterial complexity associated with skin-contact fermentation (Bouki et al., 2025; Kamilari et al., 2021). Addition, a regional microbial fingerprint may have contributed to the observed patterns, a phenomenon well documented in wines produced with minimal technological intervention (Mesnage et al., 2025).

Overall, these results suggest that the bacterial community of wine appeared to be primarily associated with the selective pressure of AF, the progression of MLF, and specific winemaking practices. These findings align with the current understanding of bacterial ecology in wine (Aires et al., 2025; Bouki et al., 2025; García-Izquierdo et al., 2024).

##### 3.1.2.1 Alpha and beta diversity of bacterial communities

**Figure 6:**
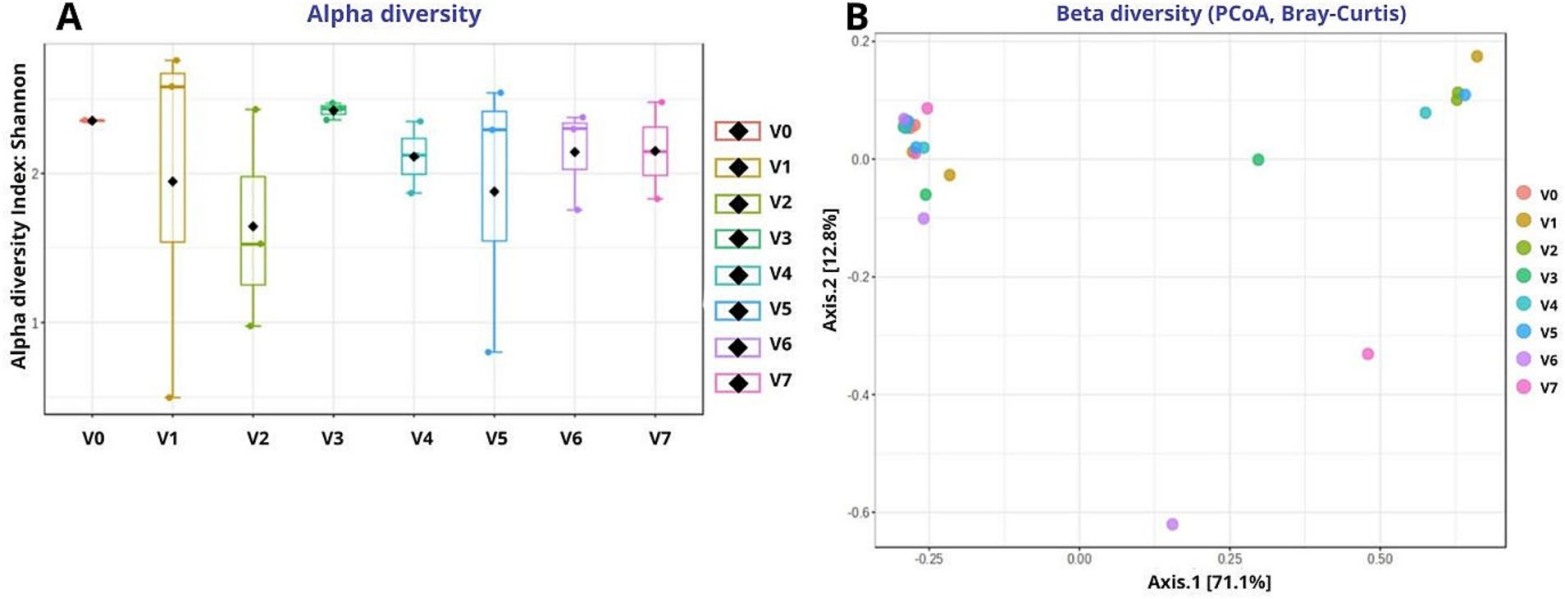
Diversity patterns of bacterial communities across different technological treatments. **(A)** Alpha diversity (Shannon index); **(B)** Beta diversity visualized by PCoA on Bray–Curtis dissimilarity. Treatments codes: V0 (Must), V1 (NS–S/S), V2 (NS–S/C), V3 (NS–S/N), V4 (S–C/S), V5 (S–C/C), V6 (S–C/N), and V7 (M–S/S). NS, Non-settled must; S, Settled must; M, Pomace fermentation; S, Spontaneous fermentation (AF or MLF); C, Controlled fermentation (AF or MLF); N, No MLF (SO_2_ 50 mg/L).

Alpha diversity analysis (Shannon index; Figure 6A) suggested comparable bacterial species richness across all technological treatments. The must samples exhibited relatively high diversity with low variability, reflecting the epiphytic bacterial microbiota of grapes, while fermented samples showed similar diversity levels across treatments. Minor compositional variations were observed at the treatment level, for example in V3, but overall, bacterial alpha diversity appeared to be associated more by the progression and timing of vinification than by observed variations across technological treatments. In line with the descriptive nature of these results, no clear divergence in species richness was attributed to the specific fermentation or MLF regimes.

Beta diversity analysis visualized through PCoA based on Bray–Curtis dissimilarity (see Figure 6B), indicated a strong overlap among bacterial communities across all technological treatments. This suggests a highly similar community composition regardless of fermentation or MLF regime applied. The observed clustering indicates that the bacterial community structure was primarily associated with the fermentation stage and sample-level variations rather than by consistent technological variations. Overall, the differences in applied technologies appeared to play a secondary role in structuring the bacterial beta diversity compared to the overarching role of the vinification process itself.

##### 3.1.2.2 Core bacterial microbiome

Core microbiome analysis across increasing abundance thresholds (see Supplementary Figure S2) suggested a progressive narrowing of the bacterial core. At the baseline thresholds (~0.01%), the core was composed of a broad assemblage of epiphytic, acetic acid, and LAB. With increasing stringency, most taxa were excluded, while *Leuconostoc* and acetic acid bacteria (*Gluconobacter*, *Acetobacter*) persisted at intermediate thresholds.

At the highest abundance thresholds (≥0.2–0.3%), the core appeared to be dominated by *Sphingomonas*, suggesting that this genus represents a consistent and relatively abundant bacterial taxon across the analyzed samples. Other taxa formed a variable and auxiliary component of the wine bacterial microbiome, likely reflecting specific technological associations and environmental variations.

##### 3.1.2.3 Abundance patterns and hierarchical clustering (heatmap analysis)

**Figure 7:**
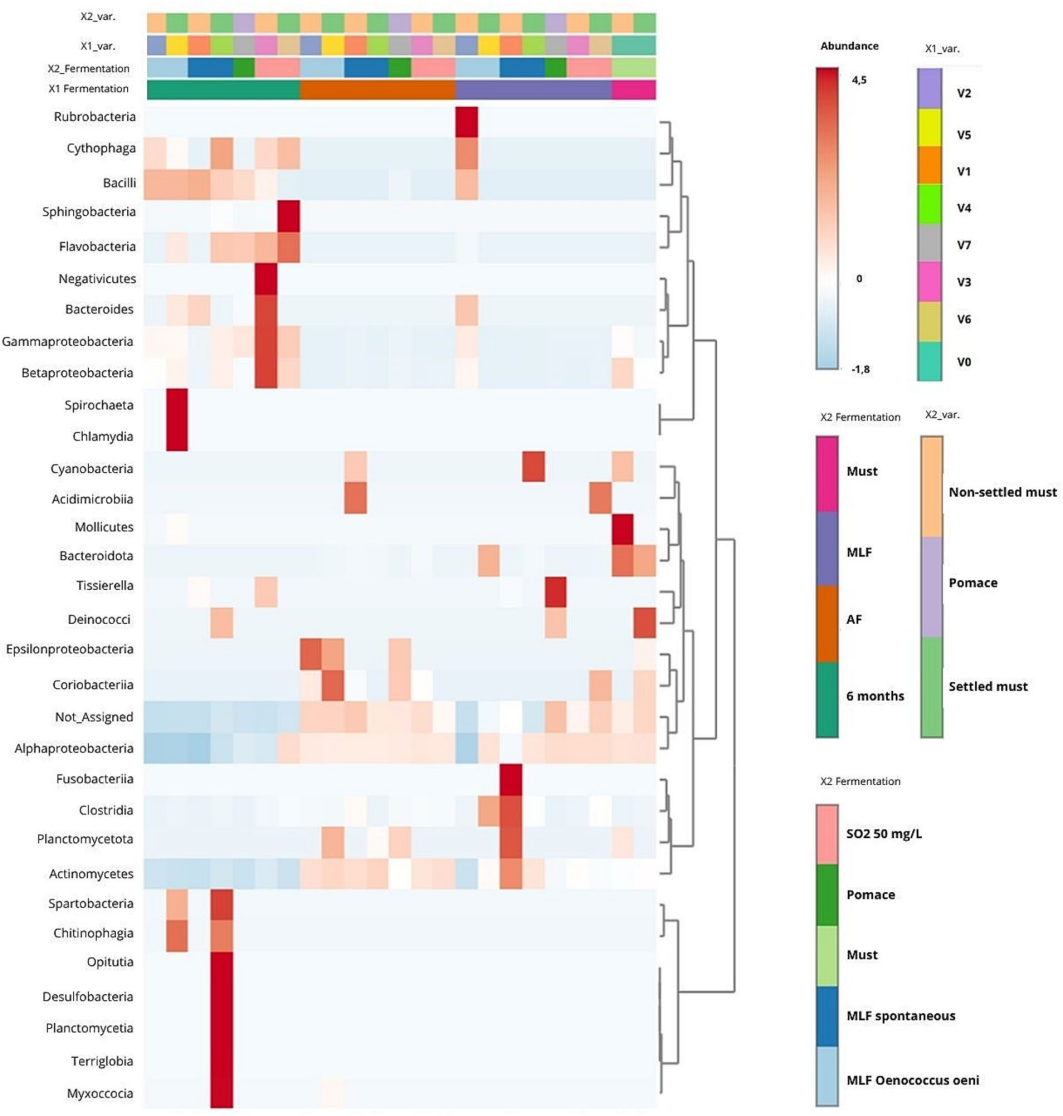
Taxonomic composition and hierarchical clustering of bacterial communities across different technological treatments. Treatment codes: V0 (Must), V1 (NS–S/S), V2 (NS–S/C), V3 (NS–S/N), V4 (S–C/S), V5 (S–C/C), V6 (S–C/N), and V7 (M–S/S). NS, Non-settled must; S, Settled must; M, Pomace fermentation; S, Spontaneous fermentation (AF or MLF); C, Controlled fermentation (AF or MLF); N, No MLF (SO_2_ 50 mg/L).

Heatmap analysis at the class level (see Figure 7) suggested that bacterial community structure followed trends primarily associated with the fermentation stage, providing a macro-taxonomic overview that complements the genus-level profiles presented in Figure 5. Must samples were dominated by epiphytic aerobic genera, whose relative abundance declined during AF. Samples collected after AF formed a cluster characterized by an assemblage of aerobic and facultatively anaerobic bacteria.

Following MLF, bacterial profiles showed divergence among treatments. Controlled MLF was associated with LAB dominance, while treatments without MLF retained communities resembling the post-AF state. After 6 months, mature wines exhibited compositionally distinct bacterial profiles, ranging from *Leuconostoc* dominance to communities enriched in acetic acid bacteria. Hierarchical clustering separated must from fermented samples, whereas compositional variations among technological treatments remained secondary. Overall, the heatmap illustrates progressive bacterial shifts during vinification, culminating in differentiated communities associated with the progression of MLF and subsequent ageing.

### 3.2 Total polyphenol content and antiradical activity

**Figure 8:**
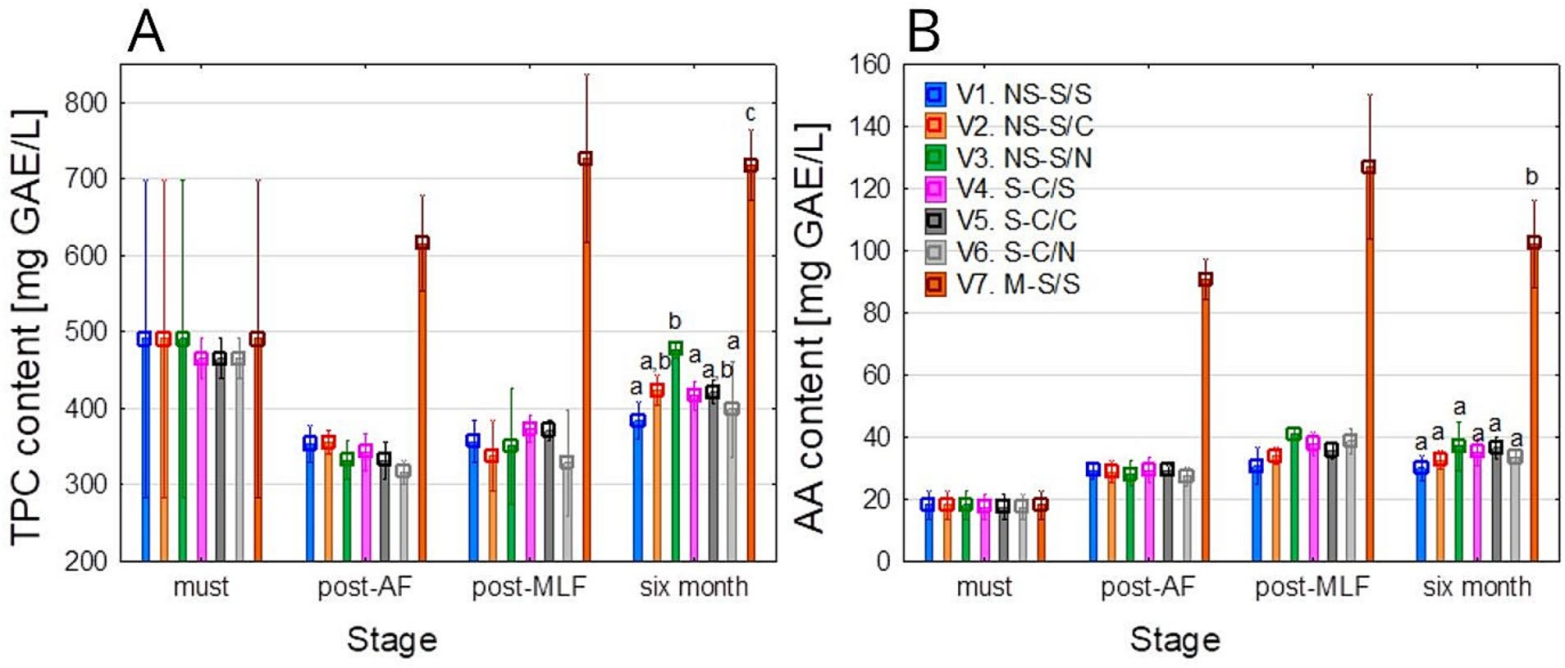
Evolution of phenolic potential across different technological treatments. **(A)** Total phenolic content (TPC); **(B)** Antiradical activity (AA). Mean values at the six-month stage were divided into homogeneous groups (letters a, b, c) using Tukey’s HSD test (*α* = 0.05); the vertical lines represent a 0.95 confidence interval. Treatment codes: NS, Non-settled must; S, Settled must; M, Pomace fermentation; S, Spontaneous fermentation (AF or MLF); C, Controlled fermentation (AF or MLF); N, No MLF (SO₂ 50 mg/L).

The factorial ANOVA showed that TPC was significantly influenced by treatment (*F* = 56.09; *p* < 0.001), stage (*F* = 27.43; *p* < 0.001), and their interaction (*F* = 4.91; *p* < 0.001). Excluding treatment V7 rendered the technological treatment (*p* = 0.232) and interaction (*p* = 0.244) non-significant, confirming that phenolic extraction intensity (skin-contact in V7) was the primary driver of the wine’s profile rather than the fermentation regime itself. Following AF, a decrease in TPC was observed in all treatments except V7 (see Figure 8A). Such decreases are commonly attributed to the adsorption of phenolic compounds by the yeast cell walls, as well as to oxidative reactions and precipitation occurring during AF (Errichiello et al., 2024; Salmon, 2006). In contrast, treatment V7 exhibited a marked increase after AF due to skin and seeds extraction (Errichiello et al., 2024; Giacosa et al., 2021). After MLF, a slight increase in TPC was observed across samples, in agreement with reports indicating that the enzymatic activity of LAB can release bound phenolic forms, thereby increasing the concentration of certain phenolic fractions (Izquierdo-Cañas et al., 2023; Wang et al., 2020).

After 6 months, V1, V4, and V6 exhibited the lowest TPC, V2 and V5 showed intermediate values, V3 displayed higher concentrations and treatment V7 clearly had the highest polyphenol levels. These results demonstrate that the overall polyphenolic profile is more strongly influenced by the composition of the raw material and the applied technology than by the type of fermentation alone. This observation is consistent with previous studies (Olejar et al., 2015; Setford et al., 2017) which have shown that technological and extractive variations between treatments often outweigh the effects of choosing spontaneous versus controlled fermentation.

In comparison with values reported in the literature, the TPC of the Sauvignon blanc wines analyzed can be regarded as relatively high. After 6 months, V1 to V6 exhibited values of approximately 380–480 mg GAE/L, whereas V7 was around 720 mg GAE/L. White wines are generally reported to TPC levels of about 100–300 mg GAE/L (Lamuela-Raventós, 2018), with some studies reporting higher values up to 220–430 mg GAE/L (Mitić et al., 2010; Mitrevska et al., 2020; Paixao et al., 2007). Studies specifically focused on Sauvignon blanc indicate a broad range of TPC, typically around 190–210 mg GAE/L for wines produced without maceration (Cáceres-Mella et al., 2014), although more detailed investigations have reported substantially higher values of approximately 340–415 mg GAE/L (He et al., 2020). From this perspective, the wines analyzed in the present study (particularly treatment V7) can be considered richer in phenols than is typically reported in the literature for Sauvignon blanc wines, which is consistent with the winemaking approach applied in this study.

Antiradical activity, determined by the DPPH assay (see Figure 8A), showed clear changes during vinification (*p* < 0.001). The factorial ANOVA revealed that when all treatments were included, the effects of technological treatment (*F* = 139.04; *p* < 0.001), vinification stage (*F* = 79.46; *p* < 0.001), and their interactions (*F* = 13.11; *p* < 0.001) were all statistically relevant. However, after the exclusion of treatment V7, the effect of technological treatment (*p* = 0.043) and the interaction (*p* = 0.047) reached only the threshold of significance, whereas the effect of vinification stage remained highly pronounced (*F* = 94.44; *p* < 0.001). This indicates that the primary sources of variability in antiradical activity were the progression of vinification and the specific extraction regime applied in V7.

In must, AA was relatively low (≈ 20 mg GAE/L), with minor changes after AF except for V7, which to approximately 90 mg GAE/L due enhanced phenolic extraction (Moreno-Arribas and Polo, 2009; Setford et al., 2017). Following MLF, AA increased slightly across treatments, with V7 exceeding 120 mg GAE/L. After 6 months, no substantial changes were observed in treatments V1–V6, with AA ranging between 30 and 40 mg GAE/L. These values fall within the range commonly reported for white wines. Mitić et al. (2010) reported antiradical activity of approximately 20–55 mg GAE/L in Serbian white grape varieties, while Mitrevska et al. (2020) described a similar range of 15–40 mg GAE/L. In contrast Paixao et al. (2007) reported very low antioxidant capacity in white wines, typically between 10 and 25 mg GAE/L. Conversely, treatment V7 exhibited substantially higher antiradical activity, around 100 mg GAE/L after MLF, which exceeded the typical values for white wines and falling within the range commonly reported for red wines (70–150 mg GAE/L) (Mitić et al., 2010; Mitrevska et al., 2020). This clear divergence highlights the critical role of extraction conditions and confirms the high phenolic potential of this treatment.

#### 3.2.1 Link between total polyphenol content and antiradical activity

TPC and AA exhibited broadly similar trends throughout vinification, largely influenced by the applied processing technology. Owing to its extensive contact with grape skins and seeds, treatment V7 consistently showed the highest TPC and DPPH values across all stages, whereas the remaining treatments remained within ranges that are typically reported for white wines. The slight increases in both TPC and AA observed after MLF are consistent with reports in the literature that describe the enzymatic release of phenolic compounds by LAB (Izquierdo-Cañas et al., 2023; Wang et al., 2020). After 6 months, descriptive differences between the treatments were most pronounced, with V7 reaching values more characteristic of red wines or skin-contact white wines. Accordingly, a very strong positive correlation was observed between TPC and AA in the final wine (Spearman’s *ρ* = 0.93, *p* < 0.05), confirming that differences in antioxidant capacity were largely related to phenolic concentration. Overall, these findings indicate that the association between TPC and AA is largely governed by extractive winemaking practices. While V7 serves as a major driver of statistical significance and represents a distinct ‘extractive system’, its inclusion allows for a clear benchmark comparison between traditional and skin-contact vinification styles.

### 3.3 Content of individual polyphenolic components

The individual polyphenolic compounds (31 monitored, 24 detected) are categorized in Table 1. This selection includes representative members from all key phenolic groups relevant to white wine, serving as reliable markers for monitoring the chemical shifts induced by the different vinification trajectories. Compounds that remained below the detection limit across all treatments are marked with an asterisk (*).

#### 3.3.1 Flavonols content

Among the flavonols, only quercetin and quercetin-3-glucoside were detected in the wines analyzed (see Supplementary Figure S3). Concentrations remained low (tens of μg/L) with no pronounced changes during vinification, except for V7, in which an increase was observed after both AF and MLF. Low flavonol levels are typical of white wines, as these compounds are predominantly found in grape skins and are only minimally extracted under standard white winemaking conditions. Previous studies have reported that white wines generally contain very low concentrations of quercetin glycosides or that these compounds are below the detection limit (Castillo-Muñoz et al., 2007; Makris et al., 2006; Scutarașu et al., 2021).

#### 3.3.2 Hydroxybenzoic acids content

Gallic, protocatechuic, and 4-hydroxybenzoic acids (see Supplementary Figure S4) were detected at all stages of vinification. Their concentrations were very low in musts, generally not exceeding 0.2 mg/L. However, a slight increase was observed in all treatments following AF. The most pronounced increase occurred in V7, in which gallic acid concentrations exceeded 4 mg/L, whereas the remaining treatments remained at levels in the order of tenths of a milligram per liter. After MLF, this gradual increase continued and after 6 months, the highest concentrations of all three acids were observed in treatment V7, with gallic acid approaching 10 mg/L. In contrast, the other treatments stabilized at moderately elevated levels relative to their initial concentrations.

The increasing trend in the level of hydroxybenzoic acids observed in the present study is consistent with previously reported findings. Olejar et al. (2015) described a marked increase in gallic and protocatechuic acids during maceration, while wines produced without skin contact typically exhibit low concentrations of these compounds. Scutarașu et al. (2021) confirmed the gradual release of hydroxybenzoic acids during fermentation and ageing and similarly reported a regular increase, particularly in technologically more extractive treatments. The substantially higher values in V7 reflect the intensified extraction typical of such treatments, consistent with findings by Boban et al. (2024) that these dynamics are governed primarily by the applied winemaking technology rather than the fermentation regime alone.

#### 3.3.3 Hydroxycinnamic acids content

**Figure 9:**
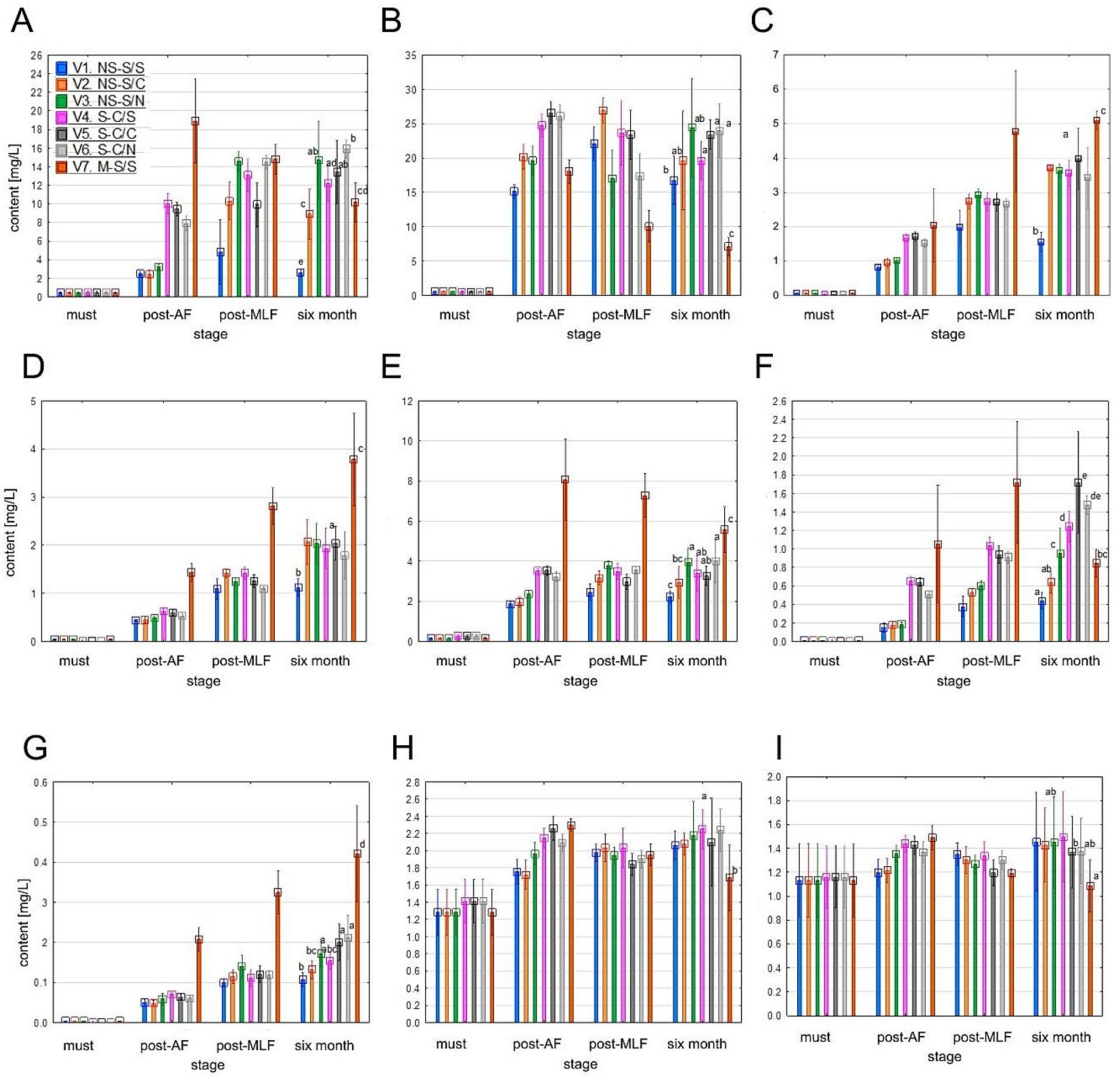
Values of individual hydroxycinnamic acids content **(A–I)**: caftaric acid **(A)**, GRP (grape reaction product – 2-S-glutathionyl caftaric acid) **(B)**, caffeic acid **(C)**, ethylcaffeate **(D)**, coutaric acid **(E)**, *p*-coumaric acid **(F)**, ethylcoumarate **(G)**, fertaric acid **(H)**, ferulic acid **(I)**. Mean values at the six-month stage were divided into homogeneous groups (letters a, b, c, …) using Tukey’s HSD test (α = 0.05); the vertical lines represent a 0.95 confidence interval. Treatment codes: NS, Non-settled must; S, Settled must; M, Pomace fermentation; S, Spontaneous fermentation (AF or MLF); C, Controlled fermentation (AF or MLF); N, No MLF (SO_2_ 50 mg/L).

Hydroxycinnamic acids were present at higher concentrations than hydroxybenzoic acids, following similar treatment trends (see Figure 9). Concentrations were low in musts but increased markedly after AF, particularly for caftaric, caffeic, and coutaric acids. Following MLF, a further slight increase was observed, likely due to the release of bound forms. After 6 months, concentrations stabilized in most treatments, whereas V7 exhibited substantially higher levels, especially of caffeic acid, ethyl caffeate, coutaric acid, and ethyl coumarate.

The observed increases during fermentation and ageing are consistent with previous reports, as hydroxycinnamic acids represent the major phenolic acids in grapes and are highly responsive to extraction intensity and enzymatic transformation. Olejar et al. (2015) demonstrated a substantial increase in caftaric and caffeic acids during maceration of Sauvignon blanc, whereas wines produced using reductive technologies typically exhibit low concentrations. Scutarașu et al. (2021) confirmed the further release and esterification of hydroxycinnamates (e.g., ethyl caffeate and ethyl coumarate) during fermentation. Yıldırım and Altındışli (2015) reported continued increases during ageing, particularly in high-phenolic like V7. Similarly, Boban et al. (2024) described higher levels in wines produced with spontaneous fermentation and enhanced phenolic extraction. More recently Eder et al. (2023) highlighted the sensitivity of caftaric acid derivatives to processing technology.

#### 3.3.4 Stilbenes content

**Figure 10:**
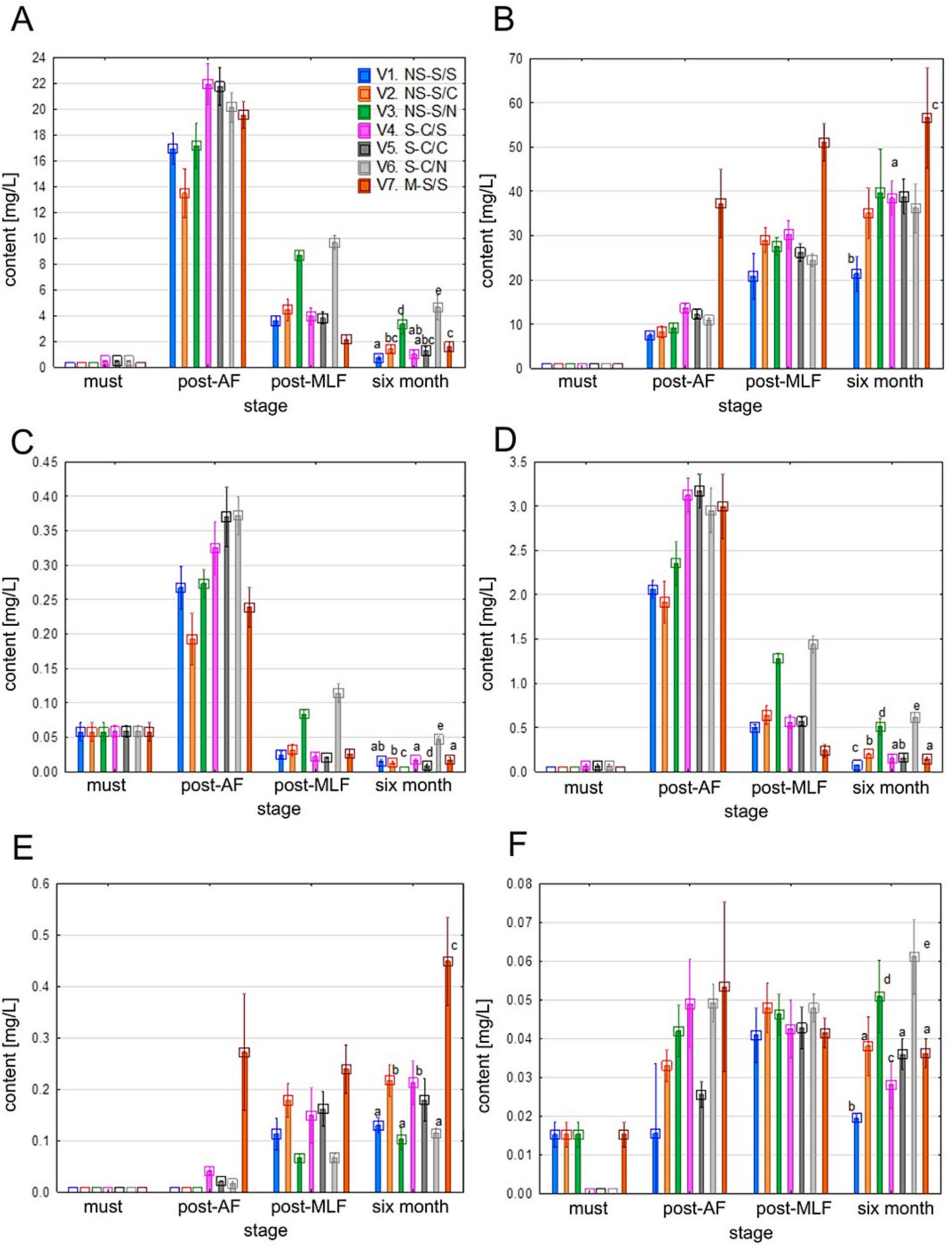
Values of individual stilbenes content. **(A–F)**: *trans*-resveratrol **(A)**, *cis*-resveratrol **(B)**, *trans*-piceid **(C)**, *cis*-piceid **(D)**, piceatannol **(E)**, astringin **(F)**. Mean values at the six-month stage were divided into homogeneous groups (letters a, b, c) using Tukey’s HSD test (*α* = 0.05); the vertical lines represent a 0.95 confidence interval. Treatment codes: NS, Non-settled must; S, Settled must; M, Pomace fermentation; S, Spontaneous fermentation (AF or MLF); C, Controlled fermentation (AF or MLF); N, No MLF (SO₂ 50 mg/L).

Stilbenes were detected in musts only at trace concentrations (see Figure 10). Following AF, their concentrations increased in all treatments, particularly for resveratrol derivatives. Most treatments showed subsequent declines after MLF and 6 months. In contrast, treatments without MLF (V3 and V6) retained higher levels of *trans*-resveratrol, *trans***−/***cis*-piceid, and astringin, suggesting that MLF contributes to their degradation. V7 showed elevated piceatannol and *cis*-resveratrol after ageing, likely due to enhanced skin extraction and gradual conversion of glycosidic forms.

Stilbenes generally occur at low concentrations in grapes and wines. Their release is strongly influenced by processing conditions. As resveratrol and its glycosides are primarily located in grape skins, white wines produced without maceration typically exhibit low stilbene levels, as reflected in the musts analyzed. The post-AF increase observed in most treatments is consistent with enzymatic conversion of piceids during fermentation (Gutiérrez-Escobar et al., 2021; Suprun et al., 2021). The decline observed during MLF and aging reflects the redox instability of stilbenes described by Benbouguerra et al. (2021),. Higher concentrations of selected stilbenes in V7 are consistent with more intensive skin extraction, as reported by Olejar et al. (2015) and Boban et al. (2024). Furthermore, previous studies (Balanov et al., 2021; Tıraş et al., 2022) have indicated that piceids and astringin belong to the relatively more stable stilbenes in white wines, which may explain their persistence after ageing. Overall, these results indicate that stilbene concentrations are primarily governed by extraction intensity and processing technology, whereas MLF mainly affects their stability and degradation.

#### 3.3.5 Flavan-3-ols content (including procyanidins)

**Figure 11:**
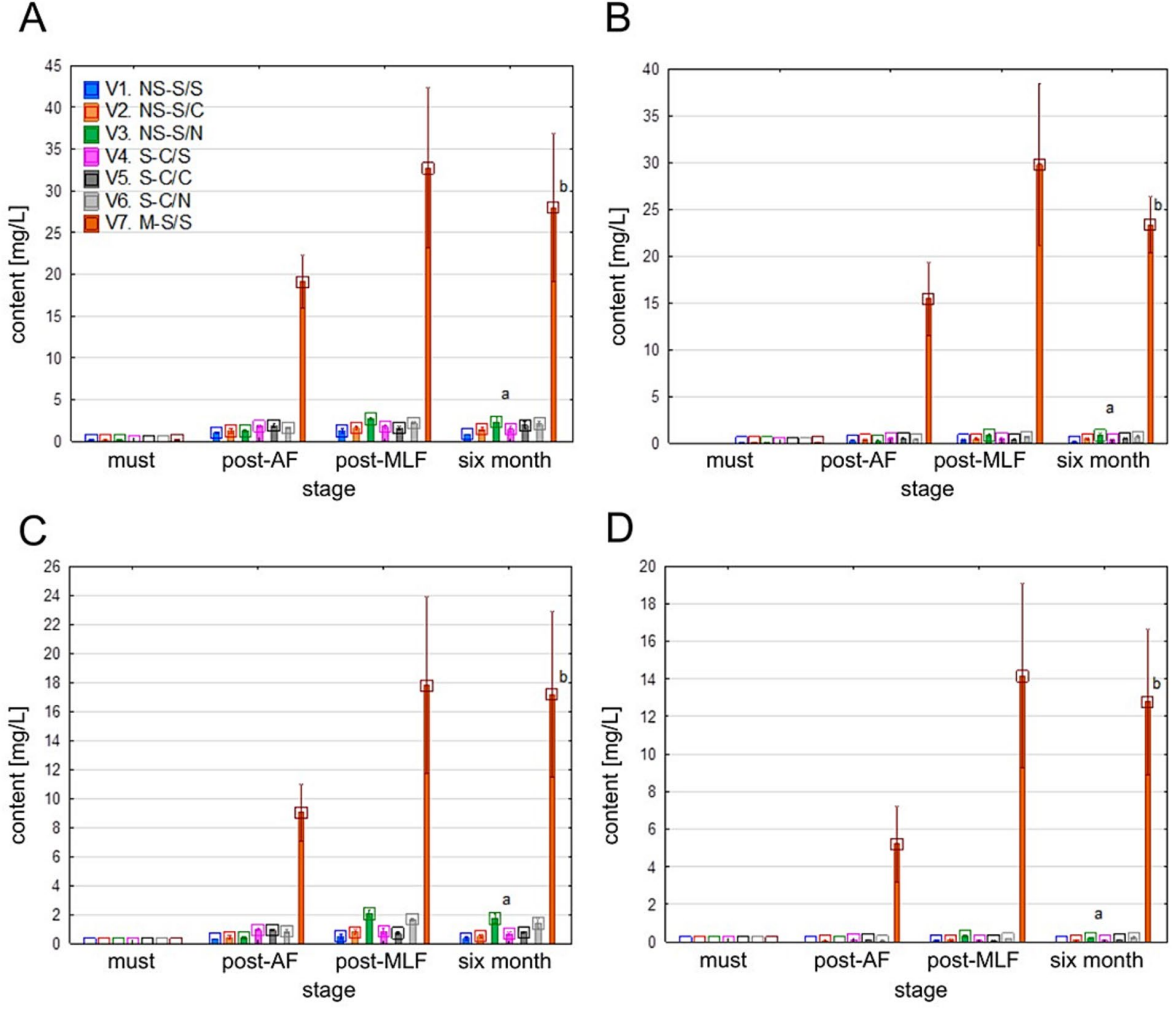
Values of individual flavan-3-ols content. **(A–D)**: catechin **(A)**, epicatechin **(B)**, procyanidin B1 **(C)**, procyanidin B2 **(D)**. Mean values at the six-month stage were divided into homogeneous groups (letters a, b, c) using Tukey’s HSD test (*α* = 0.05); the vertical lines represent a 0.95 confidence interval. Treatment codes: NS, Non-settled must; S, Settled must; M, Pomace fermentation; S, Spontaneous fermentation (AF or MLF); C, Controlled fermentation (AF or MLF); N, No MLF (SO_2_ 50 mg/L).

Flavan-3-ols were present at very low concentrations in the musts and only showed slight increases after AF and MLF in most treatments. The sole, pronounced exception, was treatment V7, in which the concentrations of catechin, epicatechin, and procyanidins increased sharply after AF to levels several times higher than those observed in the other treatments and remained markedly elevated after MLF and throughout ageing (see Figure 11).

Flavan-3-ols are major grape phenols concentrated primarily in skins and seeds, significantly influencing wine sensory properties (Liu et al., 2010; Padilla-González et al., 2022). In white wines, the combined concentration of catechin and epicatechin is commonly reported to be below 30 mg/L, with total flavan-3-ol contents typically ranging from 30 to 60 mg/L, whereas red wines generally exhibit substantially higher values (De Lima et al., 2006; Padilla-González et al., 2022). The low concentrations in V1–V6 align with standard practices, as also reported by Setford et al. (2017). In contrast, maceration or fermentation on the skins dramatically enhances the extraction of flavan-3-ols (Nicolle et al., 2021; Olejar et al., 2015; Samoticha et al., 2017; Scutarașu et al., 2021). Similar conclusions were drawn by Boban et al. (2024), who described a substantially higher phenolic extract using more extractive white winemaking practices. Since these compounds are among the most sensitive to skin-contact duration (Eder et al., 2023; Nicolle et al., 2021), V7 exhibited pronounced increases in both monomeric and dimeric procyanidins compared to non-macerated treatments.

#### 3.3.6 Main trends in the polyphenolic profile

Overall, the individual polyphenolic groups exhibited consistent trends that primarily reflected the processing technology used. While V1–V6 remained within typical white wine ranges, V7 consistently exhibited the highest concentrations across all classes due to skin fermentation. In contrast, V7 consistently showed the highest concentrations across all polyphenolic classes, in full agreement with the fact that it was the only treatment fermented directly on the pomace and therefore exhibited markedly enhanced extraction of phenolic compounds from grape skins and seeds. Treatment V7 was intentionally included in the experimental design to represent the maximum phenolic potential of the raw material. While its high phenolic load dominates the statistical variance, it provides a necessary ‘high-extraction’ reference point, allowing for a clearer differentiation between the effects of processing technology and the fermentation regime itself. The fermentation regime had a secondary influence on polyphenolic profile. Its effect was most evident for stilbenes, those treatments without MLF displayed smaller decreases during ageing, whereas for most other phenolic classes the differences between spontaneous and controlled fermentation were minor compared with the dominant effect of extraction conditions. Statistical analysis (ANOVA followed by Tukey’s HSD test) further confirmed that only treatment V7 was consistently separate from the remaining treatments, while treatments V1–V6 largely formed an overlapping homogeneous group. Collectively, these results demonstrate that the phenolic profile of Sauvignon blanc wines are primarily governed primarily by the extent of phenolic extraction during processing, with technological factors exerting a substantially stronger influence than the fermentation regime itself.

### 3.4 Interaction between the microbiome and the polyphenolic profile

#### 3.4.1 Role of phenolic compounds on fungal community succession

The development of fungal communities across the studied treatments suggests the polyphenolic composition of must and wine may be associated with the diversity of minor taxa rather than the dominance of *Saccharomyces* itself. Phenolic compounds associated with grape skins and lees have been reported to potentially affect the metabolism and adaptive capacity of early non-*Saccharomyces* yeasts, as demonstrated by Nguela et al. (2019), which aligns with the higher diversity observed in unclarified must. Nevertheless, in all treatments, *Saccharomyces* rapidly and almost completely dominated during AF, reflecting its high tolerance to phenolic stress, as previously reported by Sabel et al. (2017).

A potential link between phenolic compounds and community shifts was notable observed after MLF and during ageing, when the relative abundance of minor genera (e.g., *Hanseniaspora*, *Aureobasidium*) began to differ among treatments. This period also coincided with the greatest differentiation in polyphenolic profiles, with V7 exhibiting distinctly higher phenolic levels. Potential mechanisms for these patterns are described in studies documenting interactions between polyphenols and yeasts, including changes in cell wall properties and viability during ageing (Mazauric and Salmon, 2005) as well as the adsorption of flavanols and proanthocyanidins onto yeast cell structures (Nguela et al., 2014). However, after 6 months, treatment V4 showed higher microbial compositional heterogeneity despite low TPC, suggesting that in addition to phenolic load, other technological and ecological factors likely contribute to these shifts (e.g., the course of MLF, the microenvironment during maturation, recolonization).

#### 3.4.2 Polyphenols as selective drivers of bacterial communities

Phenolic compounds are often considered a potential factor influencing bacterial succession, particularly during MLF and subsequent ageing. Hydroxycinnamic acids, flavan-3-ols, and stilbenes are known for their inhibitory action against *Oenococcus oeni*, *Leuconostoc*, and other LAB (Dietrich and Pour Nikfardjam, 2017; García-Ruiz et al., 2008). In the present study, this trend was most notable in V7, which exhibited the highest total polyphenol content and simultaneously developed a less distinct and less stable LAB-dominated profile compared to treatments exposed to a lower phenolic load (e.g., V1 and V5).

After 6 months, the observed compositional differences between treatments were further accentuated. Treatments with a lower polyphenol content exhibited the stable dominance of *Leuconostoc*, whereas V7 maintained higher bacterial diversity, including a persistent presence of epiphytic and acetic acid bacteria. This pattern aligns with conceptual models describing how elevated phenolic load may constrain the long-term establishment of LAB while favoring phenol-tolerant taxa (Filimon et al., 2022); Sun et al. (2022). Taken together, these results suggest that polyphenols may interact with bacterial communities during MLF and ageing, potentially contributing to the observed successional pathways.

The possible selective pressure associated with polyphenols, notably observed in the delayed establishment of *Leuconostoc* in V7, may involve complex metabolic and structural interactions. High concentrations of hydroxycinnamic acids and flavanols can disrupt bacterial cell membrane integrity and inhibit key enzymatic pathways, as documented by Sabel et al. (2017). Regarding the stability of phenolic compounds, the MLF stage appears to act as a secondary modulatory phase. The slight increase in certain phenolic fractions post-MLF, as observed in our results, suggests enzymatic activity by LAB, which can release bound aglycones from their glycosidic precursors. Recent investigations into fruit-based fermentations have confirmed that LAB can modify phenolic composition and antioxidant capacity through biotransformation (Jiang et al., 2024; Zhao et al., 2023). However, the subsequent decline in stilbenes during ageing across all treatments suggests the overriding influence of redox conditions and chemical oxidation over purely microbial transformations (Benbouguerra et al., 2021). It should be noted, however, that in extractive treatments such as V7, these microbial shifts are likely associated with a combination of phenolic load and other factors, such as increased nutrient availability and solids.

#### 3.4.3 Combined effects of technology and phenolic extraction

Technological variations across the treatments were reflected in the distinct levels of phenolic extraction, which appeared to be associated with microbial succession. The most pronounced example was the pomace-fermented V7, which exhibited the highest TPC and notable microbial compositional heterogeneity during the later stages of vinification. This distinct microbial trajectory may be linked a synergistic effect: while high phenolic extraction potentially exerts selective stress, the solid phase (skins and seeds) simultaneously provides physical niches and a continuous release of nitrogenous compounds and lipids (Kamilari et al., 2021). This increased environmental complexity might partially offset the inhibitory impact of high polyphenol concentrations, potentially allowing for the persistence of a broader spectrum of bacterial taxa compared to clarified must treatments (Bouki et al., 2025). This aligns with recent findings indicating that the application of white wine lees can promote the growth of lactic acid bacteria and enhance the efficiency of MLF (Balmaseda et al., 2024). The presence of the phenol-rich environment in V7 provided an opportunity to observe microbial responses under conditions of elevated phenolic stress, suggesting that polyphenols may act as modulatory factors in bacterial succession (Dietrich and Pour Nikfardjam, 2017; García-Ruiz et al., 2008).

In contrast, treatments characterized by lower phenolic extraction (V1, V4, and V6) exhibited lower TPC after 6 months and relatively more stable bacterial profiles, frequently dominated by *Leuconostoc*. Comparisons between spontaneous and controlled MLF further suggested that inoculation with commercial starter cultures might, to some extent, counteract the potential inhibitory potential of a phenol-rich environment. In addition, must clarification reduced initial microbial heterogeneity without exerting a substantial influence on the final polyphenolic profile (Aires et al., 2025). Overall, these results suggest that microbial development was associated with the combined influence of technological interventions and phenolic availability, with polyphenols appearing to act as modulatory rather than dominant selective factors in the successional patterns during vinification.

A limitation of this study is that biological replicates were pooled prior to DNA extraction and sequencing, which does not allow assessment of within-treatment variability. Therefore, the microbiome results should be interpreted as exploratory and descriptive trends rather than strictly confirmatory. Despite this limitation, the observed trends provide valuable insight into the potential relationships between phenolic composition and microbial dynamics. Variations between treatments may also reflect underlying matrix factors (e.g., solids content, nutrient availability), which could not be fully disentangled from fermentation regime in the present design.

## 4 Conclusion

The results of this study partially support the hypothesis that winemaking technology and MLF regimes are associated with shifts in the wine microbiome and phenolic profile more significantly than the initial fermentation type (spontaneous vs. inoculated). While these trajectories proceed in parallel, the polyphenolic composition of the wines appears to be primarily influenced by technological practices, particularly the extent of contact with grape skins and seeds, whereas microbial community structure was more closely associated with the progression of fermentation and ageing.

Polyphenols did not appear to fundamentally determine the course of alcoholic fermentation but were associated with microbial successional trends during the later stages of vinification. A higher phenolic load coincided with reduced stability of lactic acid bacteria dominance and a more diverse descriptive profile of phenol-tolerant bacterial taxa. In yeasts, this potential association was mainly reflected in compositional variations among minor taxa during ageing.

Overall, technological interventions appear to provide the primary framework within which interactions between phenolic composition and microbial communities occur. Pomace fermentation was associated with a markedly distinct phenolic profile and increased compositional heterogeneity, while must clarification and controlled fermentation led to more homogeneous microbial structures. These findings suggest that polyphenols appear to act primarily as modulatory rather than determinative factors in microbial development.

Consequently, winemaking practices influencing phenolic extraction, particularly skin contact, and pomace fermentation, could potentially be explored to modulate both the chemical composition and the microbial environment of the wine. However, these microbiome results must be interpreted as exploratory and descriptive trends, as biological replicates were pooled prior to sequencing. This design precluded the assessment of within-treatment variance, and the observed shifts likely reflect a combination of phenolic load and confounding factors, such as nutrient availability from solids. Future studies incorporating independent biological replicates are necessary to confirm these preliminary relationships.

## Data Availability

All data generated in this study, including raw sequencing data and associated metadata, are available in the NCBI BioProject database under accession number PRJNA1456802.

## References

[ref1] Africa A. J. Setati M. E. Hitzeroth A. C. Blancquaert E. H. (2025). Exploring the evolution of microbial communities from the phyllosphere and carposphere to the grape must of *Vitis vinifera* L. cv’s chardonnay and pinot noir. Food Microbiol. 130:104780. doi: 10.1016/j.fm.2025.104780, 40210403

[ref2] Aires C. Maioto R. Inês A. Dias A. A. Rodrigues P. Egas C. . (2025). Microbiome and microbiota within wineries: a review. Microorganisms 13:538. doi: 10.3390/microorganisms13030538, 40142431 PMC11944700

[ref3] Andrews S. (2010). *FastQC: A Quality Control Tool for High Throughput Sequence Data*. Available online at: https://www.bioinformatics.babraham.ac.uk/projects/fastqc/.

[ref4] Aronesty E. (2013). Comparison of sequencing utility programs. Open Bioinform. J. 7, 1–8. doi: 10.2174/1875036201307010001

[ref5] Bagheri B. Bauer F. F. Cardinali G. Setati M. E. (2020). Ecological interactions are a primary driver of population dynamics in wine yeast microbiota during fermentation. Sci. Rep. 10:4911. doi: 10.1038/s41598-020-61690-z 32188881 PMC7080794

[ref6] Balanov P. E. Smotraeva I. V. Abdullaeva M. S. Volkova D. A. Ivanchenko O. B. (2021). *Study on Resveratrol Content in Grapes and Wine Products*. Paper Presented at the E3S Web of Conferences.

[ref7] Baldrian P. Větrovský T. Lepinay C. Kohout P. (2022). High-throughput sequencing view on the magnitude of global fungal diversity. Fungal Divers. 114, 539–547. doi: 10.1007/s13225-021-00472-y

[ref8] Balmaseda A. Bordons A. Reguant C. Bautista-Gallego J. (2018). Non-Saccharomyces in wine: effect upon *Oenococcus oeni* and malolactic fermentation. Front. Microbiol. 9:534. doi: 10.3389/fmicb.2018.00534, 29628914 PMC5876288

[ref9] Balmaseda A. Miot-Sertier C. Lytra G. Poulain B. Reguant C. Lucas P. . (2024). Application of white wine lees for promoting lactic acid bacteria growth and malolactic fermentation in wine. Int. J. Food Microbiol. 413:110583. doi: 10.1016/j.ijfoodmicro.2024.110583 38277869

[ref10] Bekris F. Lola D. Papadopoulou E. Vasileiadis S. Paramithiotis S. Kotseridis Y. . (2024). Spontaneous vinification supports different microbiota, volatilome and leads to wines with different sensory attributes compared to vinifications inoculated with commercial and indigenous to Vidiano cultivar *Saccharomyces cerevisiae*. LWT 205:116543. doi: 10.1016/j.lwt.2024.116543

[ref11] Benbouguerra N. Hornedo-Ortega R. Garcia F. El Khawand T. Saucier C. Richard T. (2021). Stilbenes in grape berries and wine and their potential role as anti-obesity agents: a review. Trends Food Sci. Technol. 112, 362–381. doi: 10.1016/j.tifs.2021.03.060

[ref12] Bengtsson-Palme J. Ryberg M. Hartmann M. Branco S. Wang Z. Godhe A. . (2013). Improved software detection and extraction of ITS1 and ITS 2 from ribosomal ITS sequences of fungi and other eukaryotes for analysis of environmental sequencing data. Methods Ecol. Evol. 4, 914–919. doi: 10.1111/2041-210X.12073

[ref13] Blaxter M. Mann J. Chapman T. Thomas F. Whitton C. Floyd R. . (2005). Defining operational taxonomic units using DNA barcode data. Philos. Trans. R. Soc. B Biol. Sci. 360, 1935–1943. doi: 10.1098/rstb.2005.1725, 16214751 PMC1609233

[ref14] Boban A. Milanović V. Bratinčević M. V. Botta C. Ferrocino I. Cardinali F. . (2024). Spontaneous fermentation of Maraština wines: the correlation between autochthonous mycobiota and phenolic compounds. Food Res. Int. 180:114072. doi: 10.1016/j.foodres.2024.114072, 38395560

[ref15] Bokulich N. A. Mills D. A. (2013). Improved selection of internal transcribed spacer-specific primers enables quantitative, ultra-high-throughput profiling of fungal communities. Appl. Environ. Microbiol. 79, 2519–2526. doi: 10.1128/AEM.03870-12, 23377949 PMC3623200

[ref16] Bouki P. Mitsagga C. Becker A. T. Giavasis I. (2025). *Monitoring and Characterization of the Microbiome of Organic Grapes*. Must and Natural Wine During Spontaneous Alcoholic and Malolactic Fermentation.

[ref17] Branco Z. Baptista F. Paié-Ribeiro J. Gouvinhas I. Barros A. N. (2025). Impact of winemaking techniques on the phenolic composition and antioxidant properties of Touriga Nacional wines. Molecules 30:1601. doi: 10.3390/molecules30071601, 40286197 PMC11990232

[ref18] Cáceres-Mella A. Flores-Valdivia D. Laurie V. F. López-Solís R. Peña-Neira Á. (2014). Chemical and sensory effects of storing sauvignon blanc wine in colored bottles under artificial light. J. Agric. Food Chem. 62, 7255–7262. doi: 10.1021/jf501467f 24983902

[ref19] Castillo-Muñoz N. Gómez-Alonso S. García-Romero E. Hermosín-Gutiérrez I. (2007). Flavonol profiles of *Vitis vinifera* red grapes and their single-cultivar wines. J. Agric. Food Chem. 55, 992–1002. doi: 10.1021/jf062800k, 17263504

[ref20] Chen H. Liu Y. Chen J. Fu X. Suo R. Chitrakar B. . (2022). Effects of spontaneous fermentation on microbial succession and its correlation with volatile compounds during fermentation of petit Verdot wine. LWT 168:113890. doi: 10.1016/j.lwt.2022.113890

[ref21] De Celis M. Ruiz J. Vicente J. Acedo A. Marquina D. Santos A. . (2022). Expectable diversity patterns in wine yeast communities. FEMS Yeast Res. 22:foac034. doi: 10.1093/femsyr/foac034 35862862

[ref22] De Lima M. T. R. Kelly M. T. Cabanis M.-T. Blaise A. (2006). Levels of phenolic acids, catechin and epicatechin in wines of Portugal and the Azores prodece from different varieties and vintages. Oeno One 40, 47–56. doi: 10.20870/oeno-one.2006.40.1.883

[ref23] Dietrich H. Pour Nikfardjam M. S. (2017). “Influence of phenolic compounds and tannins on wine-related microorganisms,” in Biology of Microorganisms on Grapes, in Must and in Wine, eds. König H. Unden G. Fröhlich J. (Berlin: Springer), 421–454.

[ref24] Eder R. Šćepanović R. P. Raičević D. Popović T. Korntheuer K. Wendelin S. . (2023). Study of the effects of climatic conditions on the phenolic content and antioxidant activity of Austrian and Montenegrin red wines. Oeno One 57, 68–85. doi: 10.20870/oeno-one.2023.57.3.7450

[ref25] Edgar R. C. (2013). UPARSE: highly accurate OTU sequences from microbial amplicon reads. Nat. Methods 10, 996–998. doi: 10.1038/nmeth.2604, 23955772

[ref26] Englezos V. Jolly N. P. Di Gianvito P. Rantsiou K. Cocolin L. (2022). Microbial interactions in winemaking: ecological aspects and effect on wine quality. Trends Food Sci. Technol. 127, 99–113. doi: 10.1016/j.tifs.2022.06.015

[ref27] Errichiello F. Forino M. Picariello L. Moio L. Gambuti A. (2024). Analysis of polyphenols during alcoholic fermentation of red grape Aglianico (*Vitis vinifera* L.): potential winemaking optimization and pomace valorization. Molecules 29:5962. doi: 10.3390/molecules29245962, 39770051 PMC11677095

[ref28] Fazio N. A. Albertin W. Masneuf-Pomarede I. Randazzo C. L. Caggia C. (2025). Structure of culturable indigenous yeast population and genetic diversity of Saccharomyces cerevisiae and non-Saccharomyces yeasts during spontaneous fermentation of Etna vineyards grapes. Int. J. Food Microbiol. 440:111282. doi: 10.1016/j.ijfoodmicro.2025.111282 40435560

[ref29] Filimon R. V. Bunea C.-I. Nechita A. Bora F. D. Dunca S. I. Mocan A. . (2022). New malolactic bacteria strains isolated from wine microbiota: characterization and technological properties. Fermentation 8:31. doi: 10.3390/fermentation8010031

[ref30] Garau J. Bignert M. Morata V. I. C. Merín M. G. (2025). Fungal microbiota of Malbec grapes and fermenting must under different sanitary conditions in the southern oasis of Mendoza winemaking region. Fermentation 11:553. doi: 10.3390/fermentation11100553

[ref31] García-Izquierdo I. Colino-Rabanal V. J. Tamame M. Rodríguez-López F. (2024). Microbiota ecosystem services in vineyards and wine: a review. Agronomy 14:131. doi: 10.3390/agronomy14010131

[ref32] García-Ruiz A. Bartolomé B. Martínez-Rodríguez A. J. Pueyo E. Martín-Álvarez P. J. Moreno-Arribas M. (2008). Potential of phenolic compounds for controlling lactic acid bacteria growth in wine. Food Control 19, 835–841. doi: 10.1016/j.foodcont.2007.08.018

[ref33] Garrido J. Borges F. (2013). Wine and grape polyphenols—a chemical perspective. Food Res. Int. 54, 1844–1858. doi: 10.1016/j.foodres.2013.08.002

[ref34] Ghanem C. Hanna-Wakim L. Nehme N. Souchard J. Taillandier P. El Rayess Y. (2014). “Impact of Winemaking Techniques on Phenolic Compounds Composition and Content of Wine,” in Wine Phenolic Composition, Classification and Health Benefits, ed. El Rayess Y. (Hauppauge, NY: Nova Science Publishers), 104–130.

[ref35] Giacosa S. Paissoni M. A. Río Segade S. Curioni A. Mattivi F. Piombino P. . (2021). *Phenolic Extraction and Mechanical Properties of Skins and Seeds during Maceration of Four Main Italian Red Wine Grape Varieties*. Paper Presented at the IVES Conference Series.

[ref36] González-Alonso I. Walker M. E. Vallejo-Pascual M.-E. Naharro-Carrasco G. Jiranek V. (2021). Capturing yeast associated with grapes and spontaneous fermentations of the negro Saurí minority variety from an experimental vineyard near León. Sci. Rep. 11:3748. doi: 10.1038/s41598-021-83123-1, 33580153 PMC7881026

[ref37] Granchi L. Ganucci D. Buscioni G. Mangani S. Guerrini S. (2019). The biodiversity of *Saccharomyces cerevisiae* in spontaneous wine fermentation: the occurrence and persistence of winery-strains. Fermentation 5:86. doi: 10.3390/fermentation5040086

[ref38] Gutiérrez-Escobar R. Aliaño-González M. J. Cantos-Villar E. (2021). Wine polyphenol content and its influence on wine quality and properties: a review. Molecules 26:718. doi: 10.3390/molecules26030718, 33573150 PMC7866523

[ref39] Hamaoka K. Aoki Y. Takahashi S. Enoki S. Yamamoto K. Tanaka K. . (2022). Diversity of endophytic bacterial microbiota in grapevine shoot xylems varies depending on wine grape-growing region, cultivar, and shoot growth stage. Sci. Rep. 12:15772. doi: 10.1038/s41598-022-20221-8, 36130998 PMC9492663

[ref40] He S. Hider R. Zhao J. Tian B. (2020). Effect of bentonite fining on proteins and phenolic composition of chardonnay and sauvignon blanc wines. S. Afr. J. Enol. Viticulture 41, 113–120. doi: 10.21548/41-1-3814

[ref41] Izquierdo-Cañas P. M. García-Romero E. Mena-Morales A. Gómez-Alonso S. (2023). Effects of malolactic fermentation on colour stability and phenolic composition of petit Verdot red wines. Wine Stud. 2:5795. doi: 10.4081/ws.2016.5795

[ref42] James A. Yao T. Ke H. Wang Y. (2023). Microbiota for production of wine with enhanced functional components. Food Sci. Human Wellness 12, 1481–1492. doi: 10.1016/j.fshw.2023.02.008

[ref43] Jiang J. Yin R. Xie Y. Ma X. Cui M. Chen Y. . (2024). Effects of cofermentation of Saccharomyces cerevisiae and different lactic acid bacteria on the organic acid content, soluble sugar content, biogenic amines, phenol content, antioxidant activity and aroma of prune wine. Food Chem. X 22:101502. doi: 10.1016/j.fochx.2024.101502, 38872720 PMC11170353

[ref44] Kamilari E. Mina M. Karallis C. Tsaltas D. (2021). Metataxonomic analysis of grape microbiota during wine fermentation reveals the distinction of Cyprus regional terroirs. Front. Microbiol. 12:726483. doi: 10.3389/fmicb.2021.726483, 34630353 PMC8494061

[ref45] Klindworth A. Pruesse E. Schweer T. Peplies J. Quast C. Horn M. . (2013). Evaluation of general 16S ribosomal RNA gene PCR primers for classical and next-generation sequencing-based diversity studies. Nucleic Acids Res. 41, e1–e1. doi: 10.1093/nar/gks808, 22933715 PMC3592464

[ref46] Lamuela-Raventós R. M. (2018). “Folin-Ciocalteu Method for the Measurement of Total Phenolic Content and Antioxidant Capacity,” in Measurement of Antioxidant Activity and Capacity: Recent Trends and Applications, ed. Lamuela-Raventós R. M. (Hoboken, NJ: Wiley), 107–115.

[ref47] Li H. James A. Shen X. Wang Y. (2021). Roles of microbiota in the formation of botrytized grapes and wines. CyTA J. Food 19, 656–667. doi: 10.1080/19476337.2021.1958925

[ref48] Liang L. Ma Y. Jiang Z. Sam F. E. Peng S. Li M. . (2023). Dynamic analysis of microbial communities and flavor properties in merlot wines produced from inoculation and spontaneous fermentation. Food Res. Int. 164:112379. doi: 10.1016/j.foodres.2022.112379, 36737964

[ref49] Liu Y.-X. Pan Q.-H. Yan G.-L. He J.-J. Duan C.-Q. (2010). Changes of Flavan-3-ols with different degrees of polymerization in seeds of ‘shiraz’, ‘cabernet sauvignon’ and ‘Marselan’ grapes after Veraison. Molecules 15, 7763–7774. doi: 10.3390/molecules15117763, 21060287 PMC6259140

[ref50] Makris D. P. Kallithraka S. Kefalas P. (2006). Flavonols in grapes, grape products and wines: burden, profile and influential parameters. J. Food Compos. Anal. 19, 396–404. doi: 10.1016/j.jfca.2005.10.003

[ref51] Manera C. Rivas G. A. Flores N. E. Brizuela N. S. Caballero A. C. Semorile L. C. . (2023). Prevalence of *Lentilacobacillus hilgardii* over *Lactiplantibacillus plantarum* in low-temperature spontaneous malolactic fermentation of a Patagonian pinot noir. Fermentation 9:809. doi: 10.3390/fermentation9090809

[ref52] Martins V. Teixeira A. Geros H. (2024). A comparison of microbiota isolation methods reveals habitat preferences for fermentative yeasts and plant pathogenic fungi in the grape berry. Food Microbiol. 118:104408. doi: 10.1016/j.fm.2023.104408 38049270

[ref53] Mazauric J.-P. Salmon J.-M. (2005). Interactions between yeast lees and wine polyphenols during simulation of wine aging: I. Analysis of remnant polyphenolic compounds in the resulting wines. J. Agric. Food Chem. 53, 5647–5653. doi: 10.1021/jf050308f, 15998128

[ref54] Mesnage R. Douzelet J. Seralini G.-E. (2025). Comparative analysis of fungal and bacterial composition in natural wines and their closest pesticide-treated counterparts. Sci. Rep. 15:4877. doi: 10.1038/s41598-025-88655-4 39929972 PMC11811058

[ref55] Minnaar P. Du Plessis H. Jolly N. Van Der Rijst M. Du Toit M. (2019). Non-Saccharomyces yeast and lactic acid bacteria in co-inoculated fermentations with two *Saccharomyces cerevisiae* yeast strains: a strategy to improve the phenolic content of Syrah wine. Food Chem. X 4:100070. doi: 10.1016/j.fochx.2019.100070 31656955 PMC6806450

[ref56] Mitić M. N. Obradović M. V. Grahovac Z. B. Pavlović A. N. (2010). Antioxidant capacities and phenolic levels of different varieties of Serbian white wines. Molecules 15, 2016–2027. doi: 10.3390/molecules15032016 20336029 PMC6257191

[ref57] Mitrevska K. Grigorakis S. Loupassaki S. Calokerinos A. C. (2020). Antioxidant activity and polyphenolic content of north Macedonian wines. Appl. Sci. 10:2010. doi: 10.3390/app10062010

[ref58] Mitrović D. Sredović Ignjatović I. Kozarski M. Popović-Đorđević J. (2024). Wine is more than just a beverage: chemical diversity, health benefits, and immunomodulating potential of wine polyphenols. Food Safety Health 2, 196–212. doi: 10.1002/fsh3.12036

[ref59] Moreno-Arribas M. V. Polo M. C. (2009). Wine Chemistry and Biochemistry, vol. 735 Berlin: Springer.

[ref60] Nguela J. M. Roi S. Vernhet A. (2014). *Sorption of Grape Proanthocyanidins and Wine Polyphenols by Inactivated Yeast Fractions*. Paper Presented at the 65. ASEV National Conference.

[ref61] Nguela J. M. Vernhet A. Julien-Ortiz A. Sieczkowski N. Mouret J.-R. (2019). Effect of grape must polyphenols on yeast metabolism during alcoholic fermentation. Food Res. Int. 121, 161–175. doi: 10.1016/j.foodres.2019.03.038, 31108737

[ref62] Nicolle P. Williams K. A. Angers P. Pedneault K. (2021). Changes in the flavan-3-ol and polysaccharide content during the fermentation of *Vitis vinifera* cabernet-sauvignon and cold-hardy Vitis varieties Frontenac and Frontenac blanc. Oeno One 55, 337–347. doi: 10.20870/oeno-one.2021.55.1.3695

[ref63] Nilsson R. H. Anslan S. Bahram M. Wurzbacher C. Baldrian P. Tedersoo L. (2019). Mycobiome diversity: high-throughput sequencing and identification of fungi. Nat. Rev. Microbiol. 17, 95–109. doi: 10.1038/s41579-018-0116-y 30442909

[ref64] Nilsson R. H. Larsson K.-H. Taylor A. F. S. Bengtsson-Palme J. Jeppesen T. S. Schigel D. . (2019). The UNITE database for molecular identification of fungi: handling dark taxa and parallel taxonomic classifications. Nucleic Acids Res. 47, D259–D264. doi: 10.1093/nar/gky1022, 30371820 PMC6324048

[ref65] Olejar K. J. Fedrizzi B. Kilmartin P. A. (2015). Influence of harvesting technique and maceration process on aroma and phenolic attributes of sauvignon blanc wine. Food Chem. 183, 181–189. doi: 10.1016/j.foodchem.2015.03.040, 25863627

[ref66] Padilla-González G. F. Grosskopf E. Sadgrove N. J. Simmonds M. S. (2022). Chemical diversity of Flavan-3-Ols in grape seeds: modulating factors and quality requirements. Plants 11:809. doi: 10.3390/plants11060809, 35336690 PMC8953305

[ref67] Paixao N. Perestrelo R. Marques J. C. Câmara J. S. (2007). Relationship between antioxidant capacity and total phenolic content of red, rosé and white wines. Food Chem. 105, 204–214. doi: 10.1016/j.foodchem.2007.04.017

[ref68] Paramithiotis S. Stasinou V. Tzamourani A. Kotseridis Y. Dimopoulou M. (2022). Malolactic fermentation—theoretical advances and practical considerations. Fermentation 8:521. doi: 10.3390/fermentation8100521

[ref69] Piekarska-Radzik L. Klewicka E. (2021). Mutual influence of polyphenols and Lactobacillus spp. bacteria in food: a review. Eur. Food Res. Technol. 247, 1–16. doi: 10.1007/s00217-020-03603-y

[ref70] Prathiviraj R. Rajeev R. Jose C. M. Begum A. Selvin J. Kiran G. S. (2022). Fermentation microbiome and metabolic profiles of Indian palm wine. Gene Rep. 27:101543. doi: 10.1016/j.genrep.2022.101543

[ref71] Quast C. Pruesse E. Yilmaz P. Gerken J. Schweer T. Yarza P. . (2012). The SILVA ribosomal RNA gene database project: improved data processing and web-based tools. Nucleic Acids Res. 41, D590–D596. doi: 10.1093/nar/gks1219, 23193283 PMC3531112

[ref72] Rivas G. A. Valdes La Hens D. Delfederico L. Olguin N. Bravo-Ferrada B. M. Tymczyszyn E. E. . (2022). Molecular tools for the analysis of the microbiota involved in malolactic fermentation: from microbial diversity to selection of lactic acid bacteria of enological interest. World J. Microbiol. Biotechnol. 38:19. doi: 10.1007/s11274-021-03205-0, 34989896

[ref73] Sabel A. Bredefeld S. Schlander M. Claus H. (2017). Wine phenolic compounds: antimicrobial properties against yeasts, lactic acid and acetic acid bacteria. Beverages 3:29. doi: 10.3390/beverages3030029

[ref74] Salmon J.-M. (2006). Interactions between yeast, oxygen and polyphenols during alcoholic fermentations: practical implications. LWT 39, 959–965. doi: 10.1016/j.lwt.2005.11.005

[ref75] Samoticha J. Wojdyło A. Chmielewska J. Oszmiański J. (2017). The effects of flash release conditions on the phenolic compounds and antioxidant activity of pinot noir red wine. Eur. Food Res. Technol. 243, 999–1007. doi: 10.1007/s00217-016-2817-7

[ref76] Scutarașu E.-C. Luchian C. E. Vlase L. Colibaba L. C. Gheldiu A. M. Cotea V. V. (2021). Evolution of phenolic profile of white wines treated with enzymes. Food Chem. 340:127910. doi: 10.1016/j.foodchem.2020.127910, 32882475

[ref77] Setford P. C. Jeffery D. W. Grbin P. R. Muhlack R. A. (2017). Factors affecting extraction and evolution of phenolic compounds during red wine maceration and the role of process modelling. Trends Food Sci. Technol. 69, 106–117. doi: 10.1016/j.tifs.2017.09.005

[ref78] Shimizu H. Kamada A. Koyama K. Iwashita K. Goto-Yamamoto N. (2023). Yeast diversity during the spontaneous fermentation of wine with only the microbiota on grapes cultivated in Japan. J. Biosci. Bioeng. 136, 35–43. doi: 10.1016/j.jbiosc.2023.03.013, 37088673

[ref79] Stoyanov N. Tagareva S. Yoncheva T. Shopska V. Kostov G. (2025). Significance of grape phenolic compounds for wine characteristics: dynamics and extractability during fruit maturation. Beverages 11:163. doi: 10.3390/beverages11060163

[ref80] Sun J. Ge Y. Gu X. Li R. Ma W. Jin G. (2022). Identification and characterization of malolactic Bacteria isolated from the eastern foothills of Helan Mountain in China. Foods 11:2455. doi: 10.3390/foods11162455, 36010455 PMC9407436

[ref81] Suprun A. R. Dubrovina A. S. Tyunin A. P. Kiselev K. V. (2021). Profile of stilbenes and other phenolics in Fanagoria white and red Russian wines. Meta 11:231. doi: 10.3390/metabo11040231, 33918825 PMC8070200

[ref82] Tıraş Z. Ş. E. Okur H. H. Günay Z. Yıldırım H. K. (2022). Different approaches to enhance resveratrol content in wine. Ciên. Técnica Vitiv. 37, 13–28. doi: 10.1051/ctv/ctv20223701013

[ref83] Tofalo R. Suzzi G. Perpetuini G. (2021). Discovering the influence of microorganisms on wine color. Front. Microbiol. 12:790935. doi: 10.3389/fmicb.2021.790935, 34925298 PMC8678073

[ref84] Wang S.-Y. Zhu H.-Z. Lan Y.-B. Liu R.-J. Liu Y.-R. Zhang B.-L. . (2020). Modifications of phenolic compounds, biogenic amines, and volatile compounds in cabernet Gernishct wine through malolactic fermentation by *Lactobacillus plantarum* and *Oenococcus oeni*. Fermentation 6:15. doi: 10.3390/fermentation6010015

[ref85] Waterman P. G. Mole S. (1994). Analysis of Phenolic Plant Metabolites. Hoboken, NJ: Wiley.

[ref86] Wei Y.-j. Wu Y. Yan Y.-z. Zou W. Xue J. Ma W.-r. . (2018). High-throughput sequencing of microbial community diversity in soil, grapes, leaves, grape juice and wine of grapevine from China. PLoS One 13:e0193097. doi: 10.1371/journal.pone.0193097, 29565999 PMC5863948

[ref87] Windholtz S. Miot-Sertier C. Maupeu J. Vallet-Courbin A. Lucas M. Pelonnier-Magimel E. . (2025). Influence of Sulphur dioxide management on microbial populations during wine ageing. Oeno One 59:9346. doi: 10.20870/oeno-one.2025.59.3.9346

[ref88] Wu Y. Li Z. Duan X. Dong L. Chang M. Zheng W. . (2026). Dynamic succession of the fermentation microbial community and its contribution to aroma in pomelo wine. Food Chem. Mol. Sci. 12:100371. doi: 10.1016/j.fochms.2026.100371, 41732689 PMC12925596

[ref89] Yang F. Chen C. Ni D. Yang Y. Tian J. Li Y. . (2023). Effects of fermentation on bioactivity and the composition of polyphenols contained in polyphenol-rich foods: a review. Foods 12:3315. doi: 10.3390/foods12173315, 37685247 PMC10486714

[ref90] Yıldırım H. K. Altındışli A. (2015). Changes of phenolic acids during aging of organic wines. Int. J. Food Prop. 18, 1038–1045. doi: 10.1080/10942912.2013.862631

[ref91] Zhang P. Ma W. Meng Y. Zhang Y. Jin G. Fang Z. (2021). Wine phenolic profile altered by yeast: mechanisms and influences. Compr. Rev. Food Sci. Food Saf. 20, 3579–3619. doi: 10.1111/1541-4337.12788, 34146455

[ref92] Zhao H. Liu S. Zhu L. Wang Y. (2025). Microorganisms: the key regulators of wine quality. Compr. Rev. Food Sci. Food Saf. 24:e70198. doi: 10.1111/1541-4337.70198 40421854

[ref93] Zhao X. Tang F. Cai W. Peng B. Zhang P. Shan C. (2023). Effect of fermentation by lactic acid bacteria on the phenolic composition, antioxidant activity, and flavor substances of jujube–wolfberry composite juice. LWT 184:114884. doi: 10.1016/j.lwt.2023.114884

[ref94] Živković N. M. Čakar U. D. Petrović A. (2024). Effects of spontaneous and inoculated fermentation on the total phenolic content and antioxidant activity of cabernet sauvignon wines and fermented pomace. Food Feed Res. 51, 119–129. doi: 10.5937/ffr0-50339

[ref95] Zott K. Claisse O. Lucas P. Coulon J. Lonvaud-Funel A. Masneuf-Pomarede I. (2010). Characterization of the yeast ecosystem in grape must and wine using real-time PCR. Food Microbiol. 27, 559–567. doi: 10.1016/j.fm.2010.01.006 20510771

