# Supplementary material for: Impact of winemaking technologies on polyphenolic composition and wine microbiome
Source: Front Microbiol. 2026 May 8;17:1846196. doi: 10.3389/fmicb.2026.1846196 (PMC13194491; doi:10.3389/fmicb.2026.1846196)
Supplement: Supplementary file 1 [file Table_1.DOCX]

Supplementary Material

# Supplementary Figures

**
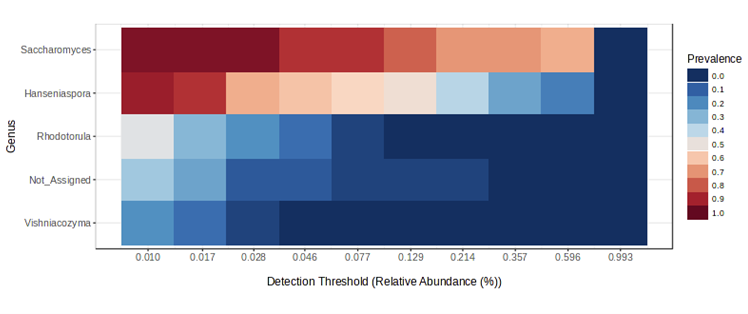
**

**Supplementary Figure S1.** Core fungal microbiome across all treatments. Core microbiome analysis based on prevalence across all treatments (V1–V7) and sampling stages, shown across increasing abundance thresholds.

**
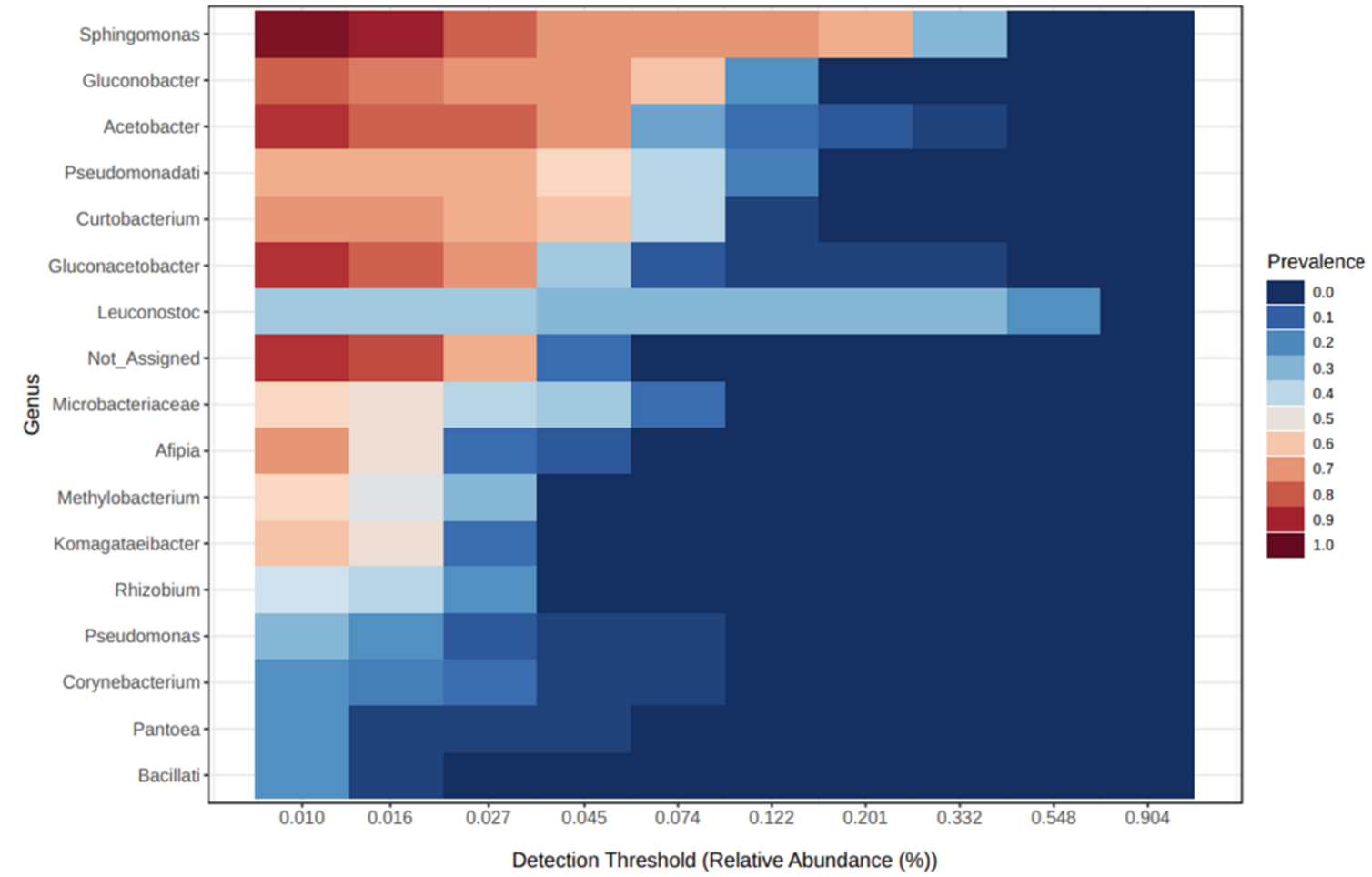
**

**Supplementary Figure S2.** Core bacterial microbiome across all treatments. Core microbiome analysis based on prevalence across all treatments (V1–V7) and sampling stages, shown across increasing abundance thresholds.


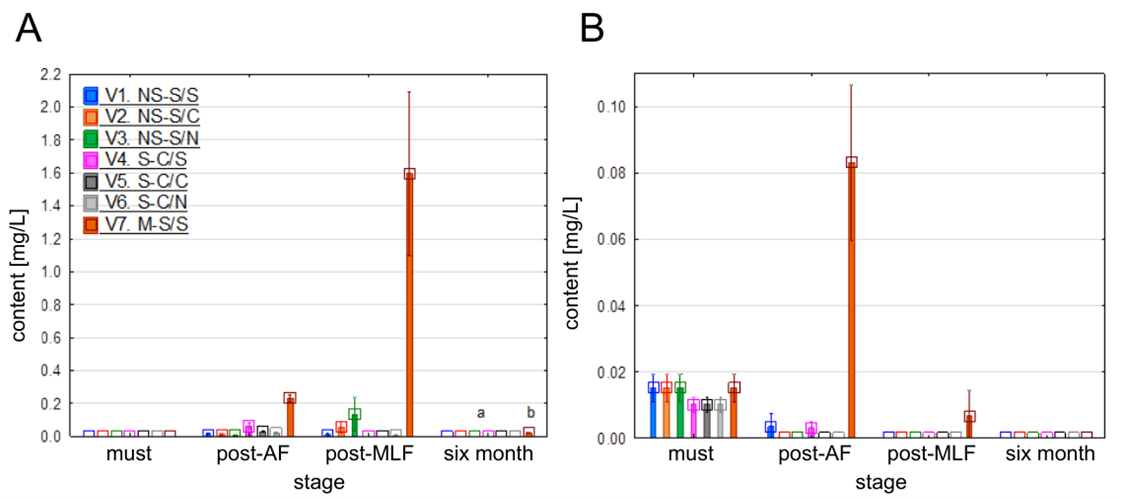


**Supplementary Figure S3.** Values of individual flavanol content A – quercetin; B – quercetin-3-glucoside; Mean values at the six-month stage were divided into homogeneous groups (letters a, b) using Tukey’s HSD test (α = 0.05); the vertical lines represent a 0.95 confidence interval. Treatment codes: NS – non-settled must, S – settled must, M – pomace fermentation; S – spontaneous fermentation (AF or MLF), C – controlled fermentation (AF or MLF), N – no MLF (SO₂ 50 mg/L).


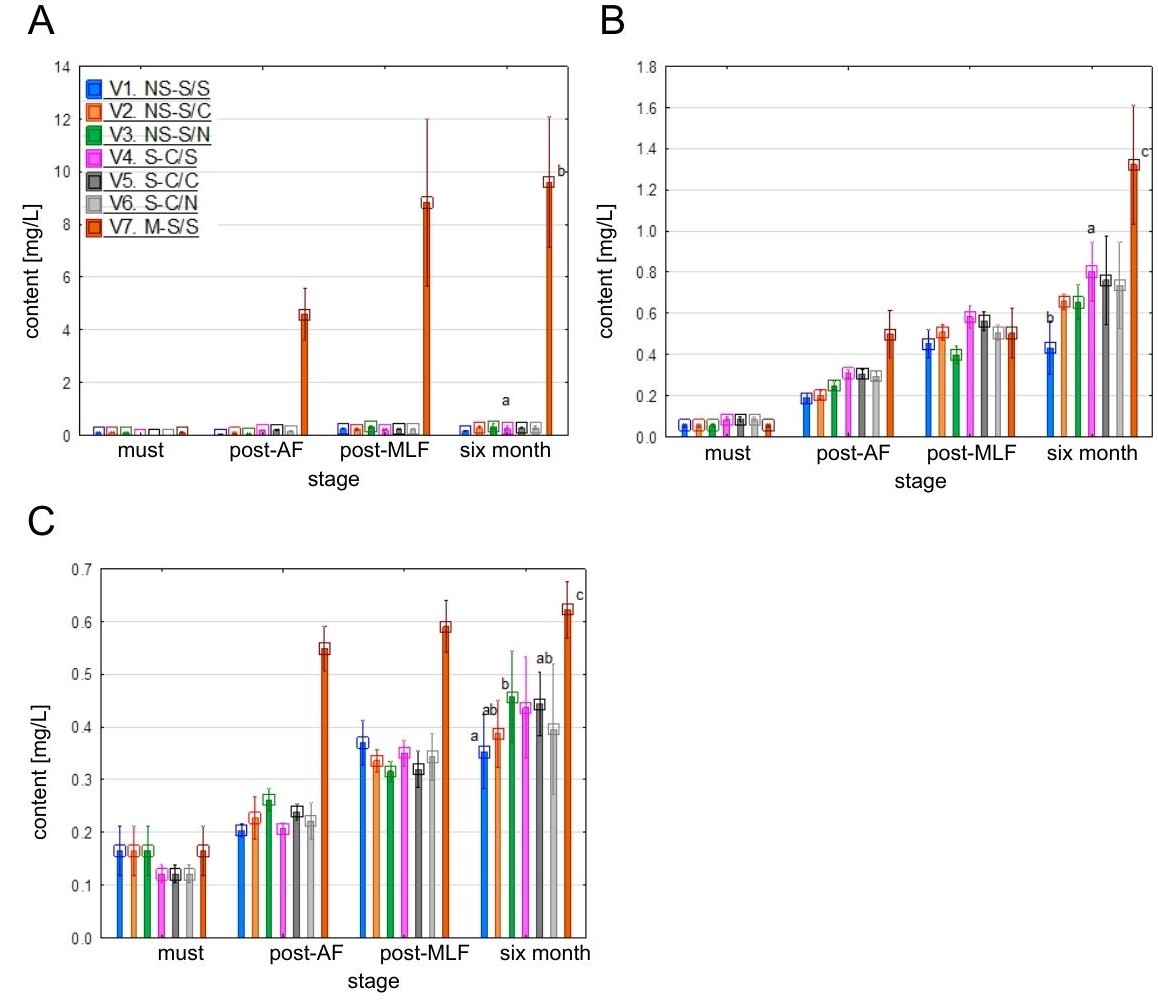


**Supplementary Figure S4.** Values of individual hydroxybenzoic acids content A – gallic acid, B – protocatechuic acid, C – 4-hydroxybenzoic acid; Mean values at the six-month stage were divided into homogeneous groups (letters a, b, c) using Tukey’s HSD test (α = 0.05); the vertical lines represent a 0.95 confidence interval. Treatment codes: NS – non-settled must, S – settled must, M – pomace fermentation; S – spontaneous fermentation (AF or MLF), C – controlled fermentation (AF or MLF), N – no MLF (SO₂ 50 mg/L).

# Supplementary Tables

**Supplementary Table 1.** MS/MS transition setting

| Q1 | Q3 | DT(ms) | Compound name | DP(V) | EP(V) | CEP(V) | CE (V) | CXP(V) |
| --- | --- | --- | --- | --- | --- | --- | --- | --- |
| 169.0 | 125.0 | 10 | Gallic acid | -42.0 | -9.2 | -5.1 | -21.9 | -0.3 |
| 153.0 | 109.0 | 10 | Protocatechuic acid | -40.3 | -9.2 | -6.8 | -21.4 | -0.1 |
| 137.0 | 93.0 | 10 | 4-Hydroxybenzoic acid | -35.4 | -8.7 | -6.8 | -22.6 | -0.3 |
| 167.0 | 152.0 | 10 | Vanillic acid | -33.0 | -9.9 | -4.9 | -20.0 | -0.6 |
| 197.0 | 123.0 | 10 | Syringic acid | -32.1 | -10.2 | -6.1 | -33.6 | -0.3 |
| 311.0 | 179.0 | 10 | Caftaric acid | -34.3 | -4.2 | -9.1 | -22.2 | -0.3 |
| 616.1 | 149.0 | 10 | GRP | -63.0 | -6.0 | -18.0 | -48.0 | -0.9 |
| 179.0 | 135.0 | 10 | Caffeic acid | -38.9 | -10.7 | -9.8 | -24.2 | -0.5 |
| 207.0 | 135.0 | 10 | Ethyl caffeate | -55.0 | -4.8 | -11.0 | -32.0 | -0.6 |
| 295.0 | 163.0 | 10 | Coutaric acid | -41.0 | -4.1 | -16.5 | -22.0 | -0.9 |
| 163.0 | 119.0 | 10 | p-Coumaric acid | -37.5 | -8.7 | -8.8 | -22.5 | -0.3 |
| 191.0 | 117.0 | 10 | Ethyl coumarate | -50.0 | -4.2 | -9.0 | -42.0 | -1.0 |
| 325.0 | 193.0 | 10 | Fertaric acid | -44.0 | -3.3 | -9.0 | -23.0 | -0.3 |
| 193.0 | 134.0 | 10 | Ferulic acid | -40.0 | -10.2 | -5.9 | -24.4 | -0.5 |
| 221.0 | 206.0 | 10 | Ethyl ferulate | -55.0 | -7.9 | -15.2 | -25.0 | -0.5 |
| 227.0 | 143.0 | 10 | Resveratrol | -70.0 | -10.6 | -11.7 | -38.2 | -0.7 |
| 389.0 | 227.0 | 10 | Piceids | -70.0 | -10.0 | -17.3 | -28.1 | -0.3 |
| 243.0 | 159.0 | 10 | Piceatannol | -70.0 | -10.0 | -7.2 | -39.1 | -0.6 |
| 405.0 | 243.0 | 10 | Astringin | -63.0 | -5.6 | -20.0 | -31.0 | -0.3 |
| 289.0 | 123.0 | 10 | Catechins | -60.0 | -9.9 | -9.5 | -44.0 | -0.3 |
| 317.0 | 151.0 | 10 | Myricetin | -74.0 | -4.0 | -9.5 | -36.3 | -0.6 |
| 301.0 | 179.0 | 10 | Quercetin | -68.0 | -7.0 | -8.2 | -29.0 | -0.3 |
| 463.0 | 300.0 | 10 | Quercetin-3-glycosides | -81.0 | -4.0 | -13.0 | -41.0 | -0.9 |
| 609.0 | 300.0 | 10 | Rutin | -93.0 | -4.3 | -17.0 | -54.0 | -0.9 |
| 285.0 | 285.0 | 10 | Kaempferol | -120.0 | -8.8 | -16.0 | -17.0 | -0.5 |
| 315.0 | 300.0 | 10 | Isorhamnetin | -74.0 | -5.0 | -19.0 | -36.0 | -0.3 |
| 447.0 | 284.0 | 10 | Kaempferol-3-D-glucoside | -78.0 | -4.2 | -12.0 | -40.0 | -0.6 |
| 441.1 | 169.0 | 10 | Epicatechin-3-Gal | -64.0 | -3.8 | -12.0 | -31.0 | -0.3 |
| 577.1 | 289.0 | 10 | Procyanidiny B | -60.0 | -4.0 | -15.0 | -37.0 | -0.7 |
| 865.2 | 289.0 | 10 | Procyanidin C | -80.0 | -7.5 | -25.0 | -55.0 | -0.6 |
| 188.0 | 93.0 | 10 | HCCA | -36.0 | -9.4 | -10.7 | -32.6 | -0.1 |
| 249.0 | 205.0 | 10 | Trolox | -58.0 | -10.0 | -7.2 | -26.6 | -0.3 |

Legend: Q1 a Q3 – parent and product ion; DT – dwell time; Mass spectrometer parameters: DP - declustering potential, EP - entrance potential, CEP - collision cell entrance potential, CE - collision energy, CXP - collision cell exit potential
